# New Dimensions in Microbial Ecology—Functional Genes in Studies to Unravel the Biodiversity and Role of Functional Microbial Groups in the Environment

**DOI:** 10.3390/microorganisms4020019

**Published:** 2016-05-24

**Authors:** Johannes F. Imhoff

**Affiliations:** GEOMAR Helmholtz-Zentrum für Ozeanforschung, Düsternbrooker Weg 20, D-24105 Kiel, Germany

**Keywords:** marine habitats, hypersaline lakes, hydrothermal vents, soda lakes, phototrophic bacteria, ammonia oxidation, denitrification, sulfate reduction, sulfur oxidation, methane oxidation

## Abstract

During the past decades, tremendous advances have been made in the possibilities to study the diversity of microbial communities in the environment. The development of methods to study these communities on the basis of 16S rRNA gene sequences analysis was a first step into the molecular analysis of environmental communities and the study of biodiversity in natural habitats. A new dimension in this field was reached with the introduction of functional genes of ecological importance and the establishment of genetic tools to study the diversity of functional microbial groups and their responses to environmental factors. Functional gene approaches are excellent tools to study the diversity of a particular function and to demonstrate changes in the composition of prokaryote communities contributing to this function. The phylogeny of many functional genes largely correlates with that of the 16S rRNA gene, and microbial species may be identified on the basis of functional gene sequences. Functional genes are perfectly suited to link culture-based microbiological work with environmental molecular genetic studies. In this review, the development of functional gene studies in environmental microbiology is highlighted with examples of genes relevant for important ecophysiological functions. Examples are presented for bacterial photosynthesis and two types of anoxygenic phototrophic bacteria, with genes of the Fenna-Matthews-Olson-protein (*fmoA*) as target for the green sulfur bacteria and of two reaction center proteins (*pufLM*) for the phototrophic purple bacteria, with genes of adenosine-5′phosphosulfate (APS) reductase (*aprA*), sulfate thioesterase (*soxB*) and dissimilatory sulfite reductase (*dsrAB*) for sulfur oxidizing and sulfate reducing bacteria, with genes of ammonia monooxygenase (*amoA*) for nitrifying/ammonia-oxidizing bacteria, with genes of particulate nitrate reductase and nitrite reductases (*narH/G, nirS, nirK*) for denitrifying bacteria and with genes of methane monooxygenase (*pmoA*) for methane oxidizing bacteria.

## 1. Introduction

The structure and functions of microbial communities are highly complex, and environmental factors as well as biological interactions are significant factors in dynamically shaping these structures. Prior to the application of molecular methods, it was almost impossible to even roughly estimate the diversity and structure of these communities. It was the application of methods based on 16S rRNA gene sequences that for the first time enabled studies on microbial diversity in environmental samples and opened our eyes to the tremendous diversity and complexity of environmental communities. 

Over the past decades, dramatic progress has been made in the knowledge and understanding on microbial diversity and function in the environment. It is fortunate to be a contemporary witness of the developments over this time, which started with first considerations on prokaryote phylogeny based on oligonucleotide catalogues of the 16S rRNA [1,2]. Soon thereafter, with the establishment of sequencing techniques in the late 1970s, complete sequences of the 16S rRNA gene became available and large data bases were established which made these sequences the primary reference for phylogenetic and systematic studies (e.g., the Ribosomal Database Project, http://rdp.cme.msu.edu/index.jsp) [3]. The sequences of the 16S rRNA gene rapidly became indispensable information for the identification of species and the phylogeny of microorganisms. Consequently, the 16S rRNA gene was also the first gene successfully applied in systematic studies on the microbial diversity of environmental communities, which might be considered as the first metagenetic studies. Despite the great enthusiasm regarding the possibility to approach microbial diversity of environmental communities, researchers soon realized that the possibilities of application of 16S rRNA gene sequences for studies of functional groups in the environment was rather limited and that functional genes were much better suited and therefore the clearly preferred choice for such studies. 

Today, the analysis of functional bacterial groups using genes of metabolic key proteins as targets has become a central topic of microbial ecology and brings a new dimension to environmental studies of microbial communities. An increasing number of investigations are concerned with diversity analysis using functional genes and receives support from the exponentially growing number of genome and metagenome sequences emerging along with the tremendously advancing sequencing technologies. These sequence data form a strong backbone for the application of functional genes in environmental studies. 

This review will focus on the application of functional genes for the analysis of environmental microbial communities and gives examples of selected functions of major ecological significance. It will be the author’s personal view on the developments during the past two decades. The diversity of selected important functional groups based on genes involved in these functions is discussed with special emphasis on marine and hypersaline habitats. Also, a few key studies related to deep sea hot vent communities are highlighted.

## 2. The Start: 16S rRNA Genes for Studies in the Environment

**Figure 1:**
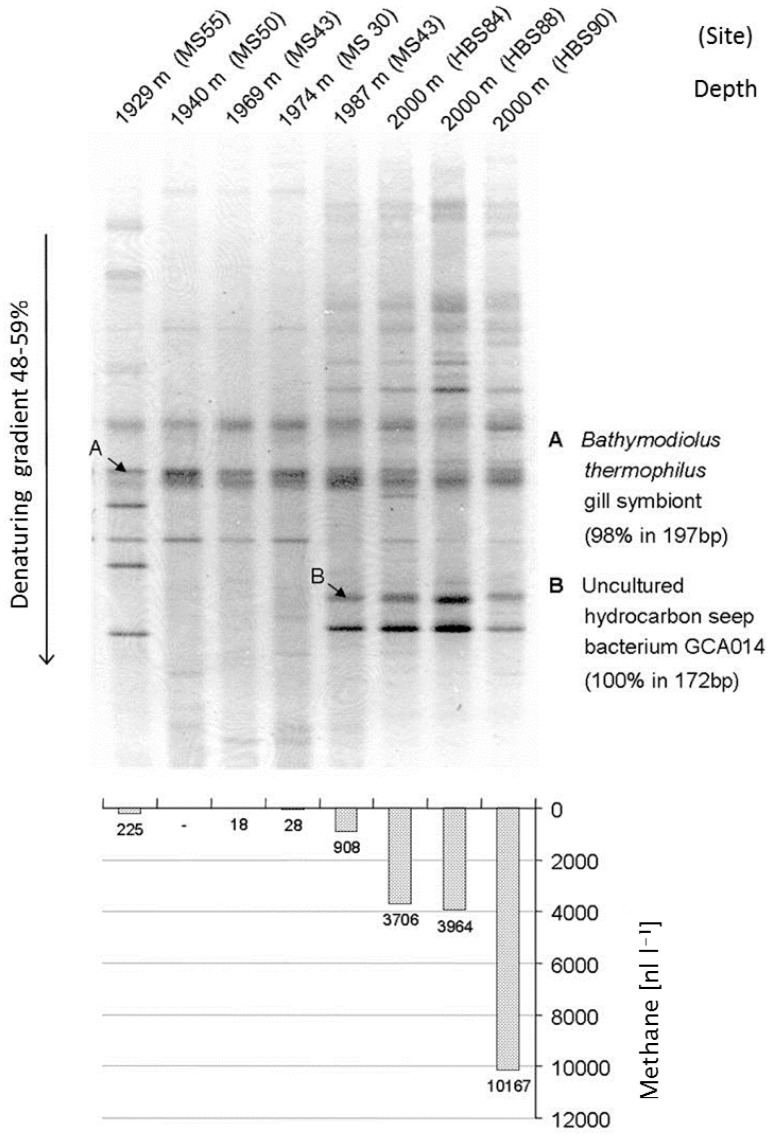
A gradient gel of amplified 16S rRNA gene sequences from deep ocean water in the vicinity of hydrothermal vents of the Fiji-Basin (**top**) and concentration of methane in the same samples (**bottom**) are shown. The band pattern clearly indicates that the presence of various strains is strongly correlated with the presence of methane in the water (from [6]).

Dramatic advances in methodology and research strategies for the analysis of prokaryote (bacterial/archaeal) phylogeny and their environmental communities have been made over the past two decades. Culture-dependent studies, the only way to approach environmental microbial communities until the 1980s, were inadequate to assess the diversity of bacterial communities. The availability of PCR techniques and extended phylogenetic trees based on 16S rRNA gene sequences from pure cultures not only enabled detailed considerations on microbial phylogeny, but also formed an initial backbone for biodiversity studies in the environment. Together with improved methods of DNA separation and purification using gel electrophoresis and cloning techniques, for the first time studies of mixed bacterial communities were possible by analysis of environmental DNA [4,5]. The favorite methods in the initial phase of these studies included separation of the mixed PCR products by gradient gels either with salt/solute gradients (denaturing gradient gel electrophoresis, DGGE) or with temperature gradients (temperature gradient gel electrophoresis, TGGE) [4,5]. These methods made possible the direct comparison of the community composition, e.g., of different samples alongside natural physicochemical gradients, from different geographic regions and from series of temporal or experimentally induced changes on a single gel. One example of such a study from the authors lab demonstrated the dependence of the community composition in the vicinity of warm deep sea vents of the Fiji Basin, in which a clear correlation was found between the presence of methane in the water samples and the bacterial community composition as revealed by 16S rRNA gene analysis using DGGE separation (Figure 1) [6]. 

However, these methods allowed identification of the bacterial species based on the 16S rRNA gene sequences only to a limited extent. In addition to biases of DNA extraction and amplification protocols, to a great part this was due to the insufficient separation of complex mixtures in the gradient gels and to the limited sequence information obtained from the short DNA stretches that yielded the best separation within the gels (approx. <500 nt). The lack of phylogenetic resolution due to the consideration of only small sequence stretches was alleviated by selection of primers that cover the complete sequence of the 16S rRNA gene, separation of the PCR products by cloning techniques and sequencing from the clone libraries [7]. These methods represented a tremendous progress in the methodology to approach environmental microbial communities at that time and caused a complete shift in research approaches and strategies. They were and still are intensively used in many laboratories worldwide.

A major drawback of the molecular approaches in microbial ecology for many years was the failure to identify microorganisms in environmental samples which was related to the discrepancies of results from culture-independent molecular analyses and culture-dependent studies. It was a general tendency that both approaches gave non-congruent views on the environmental communities (e.g., [8]). The strong selectivity of culture-based approaches on the one hand together with the really small number of microorganisms being cultured at all and the limited resolution of molecular approaches on the other hand have to be considered as major reasons for the striking discrepancies between these two approaches. Ongoing efforts to increase the cultivation success, in particular of microorganisms from so far not cultured groups, together with an improved coverage of the microbial metagenome by new sequencing techniques, is expected to alleviate this discrepancy.

Another major drawback of studies using 16S rRNA gene sequences is the failure to convincingly depict the diversity of specific functional groups in environmental communities. This is a basic problem of the 16S rRNA gene approach, as the 16S rRNA molecule is not related to metabolic properties *per se*, but only through phylogenetic observations and assumptions. Therefore, more specific approaches have to be applied in order to convincingly unravel the environmental diversity of functional groups having a unifying physiological property. This is achieved by the analysis of suitable functional genes.

## 3. Progress and Limitations of Sequence Studies of 16S rRNA Genes and Metagenomes

In view of the great enthusiasm due to the tremendous advances in environmental microbiology coming along with the use of DNA sequences for microbial biodiversity studies in the 1990s, which caused complete shifts in research approaches and initiated the field of molecular microbial ecology, the limitations of these molecular approaches appeared of minor importance. With the rigorous advances in molecular techniques over the past decade, molecular microbial ecology has grown up and analyses of the complex metagenome of environmental samples became possible. Although a steady increase in sensitivity and resolution in sequencing has been obtained, it should not be overlooked that a basic limitation in studies on the environmental metagenome has remained. This limitation is connected to the generally low number of analyzed sequences of the metagenome from a given environmental sample, with the effect that not the complete community is depicted. (The same holds for metagenetic studies that have been performed with the 16S rRNA gene as target.) If one considers that an individual sample of a marine sediment may contain more than 10^10^ bacterial cells with >10.000 different microbial species in a cm^3^ and surface waters of the oceans have an average of 1 million bacteria, a number of 100 or 1000 clone sequences of a sample (as handled in most biodiversity studies using 16S rRNA gene sequences) could cover only a minor part of the community. Only the most abundant and the most easily amplified sequences could be retrieved from the tremendous diversity of the environmental communities. Although this fraction of the more abundant organisms could be regarded to include the most important players (at least under the conditions and the time of sampling) by no means it represents the potential of a selected sample and habitat under variation of the environmental conditions. In fact, changes of the environmental conditions are expected to provoke significant and complex changes of the community composition with the effect that minor and not detected members of the community become major or even dominant parts of it [9]. Probably one of the most extreme realizations of this effect is seen in enrichment cultures used by generations of microbiologists to specifically manipulate the natural community. The design of media recipes and the definition of the culture conditions specifically select microorganisms with a desired function such as sulfate reduction, photosynthesis or methane oxidation and a desired property such as halophilic, alkaliphilic or thermophilic responses [10].

Overall, the speed of new methodological developments in sequencing technologies and the progress of knowledge in molecular microbial ecology over the past decades was unbelievable. Since the invention of PCR and sequencing techniques of the Sanger method [11,12], the most significant advances in molecular microbial ecology are realized by the high throughput sequencing technologies also called next generation sequencing technologies [13], which still continue to further improve the efficiency and performance. The first single capillary sequencer (ABI Prism 310) using the Sanger sequencing technology was on the market in 1996 and only two years later commercial 96 capillary sequencers were available [14]. This is not the place to go into details of the fascinating story of the genomic and metagenomic achievements and methodological development over the past decades. A nice overview on the development of sequencing technologies and the different platforms of high-throughput sequencing can be found in the article by Kircher and Kelso [14]. Advances in the development of microbial metagenomics, which is strongly correlated to the progress in sequencing technologies, are nicely summarized by Gilbert and Dupont [15].

Sequencing technologies made possible the sequence analysis of the first bacterial genome in 1995 (*Haemophilus influencae*, 1.8 Mb) [16]. At that time, this was a great effort made possible by use of the Sanger method, today this amount of sequence information can be obtained within hours from the new high throughput sequencing platforms [14]. For comparison, the sequencing throughput with the well-established Sanger method now is at approx. 6 Mb/day (three-times the genome size of *Haemophilus influencae*), while the throughput of the Illumina technology and similar other technologies is at 5000 Mb/day (almost 2800 times the genome size of *Haemophilus influencae*, more than 1000 times the genome size of *Escherichia coli* and approx. 1.5 times the human genome size) [14]. These are impressive numbers and it is interesting to compare them with the sequencing efforts of metagenomic studies and the size of environmental metagenomes. In a great effort published in 2004, microbial metagenomes in the Sargasso Sea were studied with the Sanger technology resulting in the production of 1 billion non-redundant base pairs [15,17]. This number compares to 1 million bacteria present within 1 mL sea water and an average content of 5 million base pairs per bacterium, *i.e.*, an information content of 5 × 10^12^ (5 trillion) base pairs in 1 mL, 10^15^ base pairs per Liter and 10^18^ base pairs per 1000 L, with the implication that metagenomic studies before 2010 have used sample volumes from 1–1000 L sea water [15]. With a further thousand-fold increase of the sequencing effort of the high-throughput technology on a daily basis from 5000 Mb/day and considering a sampling size of only 1 L (equivalent to 10^15^ base pairs of the metagenome), the coverage would be 0.1%. These numbers reflect both, the incredible advances in sequencing technologies on the one hand and the immense complexity of microbial metagenomes on the other hand. At this point it is necessary to recall the breathtaking improvements in the handling of data by computing facilities and software programs over the past 2 decades that all of us have witnessed in the daily life. Without special efforts and specific software programs, the handling of sequence information of a complete metagenome containing 1 billion single prokaryote genomes would not be possible. The evaluation of metagenomic data by bioinformatics tools is another great challenge to metagenome analysis [15]. 

## 4. Functional Gene Studies

Because much of the motivation to study bacterial and microbial biodiversity is coming from the desire to understand the functions of the environmental communities and the interactions within these communities, it was a logic step to include functional genes into biodiversity studies and to study functionally defined fractions of the whole microbial community. Functional gene approaches to a great part overcome limitations of resolution and in particular of lacking specificity in microbial community analyses by the 16S rRNA gene approach. They have a number of advantages for investigations of environmental communities. First of all, these approaches focus their view on a selected functional aspect of the environment and on organisms involved in this specific function. Second and most important, these approaches achieve a better resolution regarding the community composition, because the number of genes of a particular function in a given sample is much smaller compared to the number of 16S rRNA genes (and whole genomes) and (assumed that the used primer system not only is specific to the target gene but also covers the diversity of this gene in the widest possible range) less abundant strains may be much easier detected and ideally the whole functional community can be resolved. In addition, many functional genes show faster rates of evolution relative to the 16S rRNA gene, which specifically has been selected to trace prokaryote phylogeny because of its phylogenetic conservative properties [1,2]. In consequence, their sequence comparison results in a higher phylogenetic resolution, supposed that specific and informative sequence stretches are selected for the analyses. In particular, closely related microorganisms within a defined functional group often can be more easily distinguished by functional genes compared to the 16S rRNA gene. Therefore, functional gene approaches are excellent tools to study the diversity of a particular functional group and to demonstrate changes in the composition of prokaryote communities contributing to the selected function upon natural or experimentally induced changes of the environmental conditions [9,18]. 

The main challenges at the initiation of functional gene approaches in the late 1990s included the selection of suitable functional genes, the design of appropriate primer sets to amplify sequence stretches of sufficient length and information as well as the establishment of databases with sequences of these genes in order to allow phylogenetic assignment and eventually identification of the players in the environment. Today, it is hardly imaginable, that the lack of appropriate gene sequences in the databases was a major limitation at the initial stages of such investigations and even made it difficult to design proper primer sequences to specifically approach the target gene [8]. 

The selected genes and the established primers on one hand need to be specific for the selected metabolic pathway on the other hand the primers/selected sequence stretches should enable a complete consideration of the biodiversity of the selected pathway and function. In addition, the selected sequences should contain sufficient phylogenetic information and ideally contain more than 1000 nucleotides to enable good phylogenetic resolution. For a number of functional genes, appropriate primer systems have been described (see Table 1) and databases of functional gene sequences rapidly grow up, in particular due to the growing number of complete genome sequences and the increased potential in performing metagenetic analyses with functional genes.

Although lateral gene transfer may be relevant for most if not all functional genes and potentially may blur the phylogenetic signal as compared to that of the 16S rRNA gene, experience with quite a number of functional genes and the comparison of their phylogeny with that of the 16S rRNA makes clear that events of lateral gene transfer were not frequent events [19,20,21,22,23,24]. They can be localized and do not limit the application of functional genes in diversity studies and in the identification of species in the environment. In consequence, advanced phylogenetic trees of functional genes can establish a functional phylogeny, independent from 16S rRNA phylogeny, and functional gene sequences enable species identification in the environmental metagenome with increasing confidence, as outlined below with examples of the anoxygenic phototrophic bacteria.

In general, most eco-physiological key functions are found in various phylogenetic lineages and in addition multiple pathways may exist for the same biogeochemical function, as for instance for the oxidation of sulfur compounds, ammonia oxidation, methane oxidation and autotrophic carbon dioxide fixation. The different pathways of the same biochemical function also present different target genes for the molecular analysis. This necessitates the design of different primer systems for each of the target genes of a particular function considered for community analysis, but at the same time it provides the advantage to distinguish the different groups of the studied function. In addition, the same genes may be involved in different functional roles, as exemplified by adenosine-5′phosphosulfate (APS) reductase (*ap*r) and dissimilatory sulfite reductase (*dsr*) playing roles in sulfate reduction as well as in sulfur oxidation [19,20,25]. Furthermore, different functional reactions may have common evolutionary roots and show strong sequence homologies of their genes, as known for methane monooxygenase (*pmo*) in methane-oxidizing bacteria and ammonia monooxygenases (*amo*) in ammonia-oxidizing bacteria [26].

The presence of a particular target gene and the sequences thereof yield valuable information on the organisms involved in this function in a particular habitat, on their phylogenetic relationships and eventually on their identity. If a comprehensive database is available on functional target gene sequences from identified type cultures, even species identification of the participating microorganisms may be possible [9]. With these implications, functional gene analysis is perfectly suited to link culture-based microbiological work with environmental molecular genetic studies. Major goals on the future perspective for such studies are to achieve not only the function-specific diversity but enable species identification in environmental communities, relate specific functions to microbial species or phylotypes, determine their habitat preference and environmental adaptation as well as their biological activity based on functional transcriptome analyses.

To approach the functional diversity in environmental systems today two strategies are available, (i) the analysis of whole metagenomes and (ii) the selective analysis of functional genes. Even if the coverage of the whole metagenome is small, a tremendous amount of information is becoming available from metagenomic studies, which includes information on functional genes. Thus metagenome studies will continue to provide valuable information for functional aspects in the environment. However, for the specific functional analysis of a particular microbial community, the efforts of metagenomic analysis are not required and the coverage of the functional gene sequences is expected to be much better using up-to-date sequencing techniques for amplicons of specific functional genes. The major advantages of functional metagenetic approaches are the high specificity and the use of a comparable small data volume of sequence information to be analyzed and evaluated.

In order to specifically study communities of functional microbial groups and their responses to environmental factors, genetic tools were established for various important eco-physiological functions. Examples are presented with the following target genes/proteins: for two types of anoxygenic phototrophic bacteria, the Fenna-Matthews-Olson-protein (*fmoA*) as target for the green sulfur bacteria and two reaction center proteins (*pufLM*) for the phototrophic purple bacteria, adenosine-5′phosphosulfate (APS) reductase *(aprA*), sulfate thioesterase (*soxB*) and dissimilatory sulfite reductase (*dsrAB*) for sulfur oxidizing and sulfate reducing bacteria, ammonia monooxygenase (*amoA*) for nitrifying/ammonia-oxidizing bacteria, nitrate and nitrite reductases (*narH/G, napA, nirS, nirK*) for denitrifying bacteria and methane monooxygenase (*pmoA*) for methane oxidizing bacteria (see Table 1 for recommended primers). In addition, a few studies are highlighted in which multiple functional genes have been used to characterize the potential of deep sea hot vent communities.

## 5. Photosynthesis and Anoxygenic Phototrophic Bacteria

Anoxygenic phototrophic bacteria are major players in a number of ecological niches, which primarily are strictly anoxic but extend to microoxic and even oxic environments [27,28,29,30,31]. We distinguish several major phylogenetic lines as well as different physiological groups which occupy defined and different niches in freshwater, marine and hypersaline environments [29,30,32]. These lines are represented by members of different bacterial phyla, by the green sulfur bacteria or Chlorobiaceae (Chlorobi) [33], the Heliobacteriaceae (Firmicutes) [34], the green nonsulfur bacteria or Chloroflexaceae [35], the Chloroacidobacteria [36,37], and the phototrophic purple bacteria (purple sulfur and purple nonsulfur bacteria), which are Alpha-, Beta- or Gammaproteobacteria [38,39,40,41]. The so-called aerobic phototrophic purple bacteria, at least most of those known to date, are close relatives of the purple nonsulfur bacteria, in particular of the Alphaproteobacteria (though representatives of Beta- and Gammaproteobacteria are known as well) close to *Rhodobacter* and *Rhodovulum* species, have a primarily chemoheterotrophic metabolism and are aerobic respiring bacteria containing bacteriochlorophyll [31,42,43]. In metagenetic studies using short *pufM* sequences, they have been recognized as important players in the open ocean [44,45] and also in brackish water lagoons [46]. In the following, we will focus on the phototrophic green and purple sulfur bacteria, because systematic and diversity studies of these groups are most advanced.

Pitfalls of applying 16S-rRNA-based approaches to the analysis of communities of anoxygenic phototrophic bacteria, in particular the phototrophic Proteobacteria (the purple sulfur and nonsulfur bacteria), were due to the close phylogenetic relationship between phototrophic and nonphototrophic bacteria within the Proteobacteria. In consequence, specific sequence stretches of 16S rRNA genes that would clearly allow the design of specific primers and the identification of phototrophic representatives in complex mixtures and environmental samples could not be identified. This necessitated the use of functional genes, e.g., those related to photosynthesis to study the environmental diversity of these bacteria. 

In order to specifically analyze communities of anoxygenic phototrophic bacteria and their adaptation to different environmental conditions and their geographic distribution, primer systems have been developed that specifically target these bacteria: the *fmoA* gene encoding for a bacteriochlorophyll-a protein specific for the green sulfur bacteria and Chloroacidobacteria [47] and the *pufLM* genes encoding for reaction center proteins of the bacterial photosystem II which is specific for all phototrophic purple Proteobacteria and Chloroflexaceae [9,48]. 

Important steps for the possible identification of species in environmental DNA sequences were the establishment of a phylogenetic-based taxonomy supported by 16S rRNA gene sequences and the demonstration of a general congruence of the phylogenies of 16S rRNA genes with those of *fmoA* and *pufLM* genes [49,50,51,52,53,54]. The formation of a comprehensive database of *fmoA* genes of green sulfur bacteria [47] and of *pufLM* genes of purple sulfur bacteria [48] from most cultured reference and type strains enabled detailed studies of environmental communities of these bacteria and the recognition of genera and species in the natural habitat. 

### 5.1. The Phylogeny of the fmoA Gene in Green Sulfur Bacteria

The FMO protein is a bacteriochlorophyll-a protein that mediates energy transfer between the chlorosomes and the reaction center in the cytoplasmic membrane of green sulfur bacteria [55] and the recently described phototrophic thermoacidophilic *Chloroacidobacterium thermophilum,* which occupies special ecological niches in hydrothermal springs and belongs to the Acidobacteria phylum [36,37]. FMO is absent in another major phylogenetic line of phototrophic green bacteria containing chlorosomes, the Chloroflexi [56]. Therefore, *fmoA* is an appropriate target to specifically analyze environmental communities of the green sulfur bacteria [57] and phototrophic Chloroacidobacteria. A comprehensive phylogeny of the green sulfur bacteria, based on phylogenies of both 16S rRNA and *fmoA* gene sequences was established including available type strains of the established species [47]. Remarkably, the phylogenies of the two independent genes were largely congruent and species and strains can be easily well identified by either of the two genes. The available information on sequences from both *fmoA* and 16S rRNA genes was used in the taxonomy of these bacteria to rearrange the strains and species, to redefine some species and in a few cases establish new species and a new genus [54]. With this background, environmental communities of green sulfur bacteria can be resolved on the species level by *fmoA* sequence analyses [58].

### 5.2. The Phylogeny of the pufLM Genes in Purple Sulfur Bacteria

In order to selectively approach the phylogeny of the phototrophic purple bacteria and to develop tools for the analysis of natural communities of these bacteria, the *pufLM* genes were selected, which code for the light (L) and medium (M) subunit of the photosynthetic reaction center type II structural proteins of all phototrophic Proteobacteria (purple sulfur bacteria, purple nonsulfur bacteria as well as the aerobic phototrophic purple bacteria producing bacteriochlorophyll and forming a photosynthetic apparatus) and in addition of the phototrophic members of the Chloroflexi [48]. An improved primer system was designed covering a large part of the combined *pufL* and *pufM* genes and in addition, a comprehensive database of *pufLM* gene sequences of most of the recognized type strains of the purple sulfur bacteria was established [48]. The phylogenetic relationship demonstrated by *pufLM* gene sequences of the purple sulfur bacteria (Gammaproteobacteria) was in good correlation to that of 16S rRNA gene sequences [48]. A phylogenetically based taxonomy of the purple sulfur bacteria facilitated the sequence-based species recognition in environmental samples [52,53,59]. Thus, the *pufLM* genes qualified as a valuable tool for studies of environmental communities of the phototrophic purple bacteria allowing species recognition of these bacteria even in complex mixtures of environmental communities.

### 5.3. Molecular Ecology and Species Recognition of Phototrophic Sulfur Bacteria in the Environment

Sequence information is predestined to link bacterial systematics and functional ecological studies because (i) sequence information is now well established as a property in bacterial systematics and forms a backbone for bacterial phylogeny and systematic treatment; and (ii) sequence information becomes easily available from environmental communities, from individual clones as well as from complete metagenomes and can contain important information about the physiological potential at the level of individual strains and species. Environmental gene sequences (or rather the bacteria associated with the sequences) can be arranged in phylotypes, which comprise sequences above a defined similarity. If a distinction of phylotypes is made at a sequence level that compares to the level of distinction between species with pure cultures, phylotypes can be used to approach the species diversity of environmental communities. If environmental clone sequences are sufficiently similar to known species, represented by their type strains, it is quite likely that they are representatives of this species or close relatives thereof. If considerations concerning sequence similarities as a rough guide for species differentiation of pure cultures are transferred to sequences from the environment, species recognition and an estimate of the species diversity in environmental samples can be achieved with phylotypes as an equivalent to the taxonomic defined species. Similar considerations as have been proposed as guidelines for the use of 16S rRNA gene sequences in taxonomy can be made for functional genes [60]. With the consideration of evolutionary rates in comparison to the 16S rRNA gene, borderlines of 86% and 95% sequence similarity of the *pufLM* genes have been proposed for the distinction of genera and species, of purple sulfur bacteria, respectively [9,48].

### 5.4. Environmental Communities of Green Sulfur Bacteria

Phototrophic green sulfur bacteria are common in coastal lagoons and marine sediments, where they often form massive colored mass developments, while in freshwater lakes their development is mostly hidden in the depth at the chemoclines. They are dominant primary producers in the Black Sea [61] and develop in other stratified ocean basins [62]. They have the most advanced antenna system to harvest light and accordingly are adapted to life at minute amounts of light [63].

**Figure 2:**
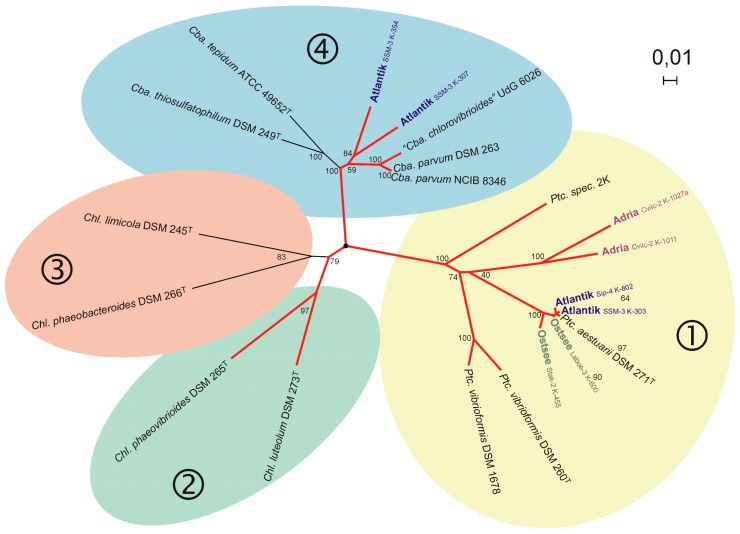
The phylogenetic relationship of marine green sulfur bacteria (connected with red lines) according to *fmoA* sequences (according to [57]) is shown. Reference strains and type strains of known species are indicated and environmental sequences are highlighted and originate from the Croatian Lake Malo Jezero (“Adria”), a Baltic Sea lagoon (“Ostsee”) and Sippewissett salt marsh USA (“Atlantik”). It highlights the phylogenetic relation of green sulfur bacteria of the *Prosthecochloris* group from different geographic locations. Strain 2K represents the branch including *Prosthecochloris indica* [64].

With the comprehensive study of *fmoA* and 16S rRNA gene phylogenies of pure cultures of green sulfur bacteria as a solid basis, a first detailed molecular genetic study on the species composition of green sulfur bacteria communities was made with samples from marine and saline habitats of different geographical locations, of the Baltic Sea, the Mediterranean Sea, Sippewissett salt marsh (MA, USA) and Bad Water (Death Valley, CA, USA) using the established primers [57]. Quite interestingly, all of the clone sequences (more than 370 16S rRNA gene sequences and more than 130 *fmoA* sequences) were associated with salt-dependent phylogenetic lines of green sulfur bacteria which had been previously established with pure cultures [47]. The clear dominance of representatives of the true marine green sulfur bacteria, in particular of the genus *Prosthecochloris* [47,54] was in support of the experience from culture-dependent approaches, which regularly yielded *Prosthecochloris aestuarii* in enrichments and as isolated cultures from many marine habitats (Figure 2) [32]. 

Though culture-based studies always had left doubt whether the specific cultivation conditions had selected just strains of *Prosthecochloris* from a more complex environmental community, the genetic analyses conclusively demonstrated that members of *Prosthecochloris* are the dominant green sulfur bacteria in suitable marine and saline habitats. Apparently, the phylogenetic diversity of marine green sulfur bacteria belonging to *Prosthecochloris* is significantly higher than was known from pure cultures. Available sequence information allowed the recognition of at least four different groups within this genus which are likely to represent different species [57], including the established species *Prosthecochloris aestuarii*, *Prosthecochloris vibrioforme* and *Prosthecochloris indica* [54,64] and additional phylogenetic lines represented by environmental sequences [57,65].

### 5.5. Environmental Communities of Phototrophic Purple Bacteria

Phototrophic purple sulfur bacteria are Gammaproteobacteria and are forming massive colored blooms in coastal sediments and lagoons and regularly are accompanied by green sulfur bacteria and purple nonsulfur bacteria (Alphaproteobacteria and Betaproteobacteria) [32,62]. In the coastal zone the phototrophic bacteria are exposed to considerable changes in salinity, temperature and other parameters (in particular concentrations of oxygen and sulfide forming countercurrent gradients), which are expected to significantly shape and modify the composition of these communities. Adaptation to these changing conditions is prerequisite for the successful competition of the species and differential adaptation determines the dominance of different species alongside the natural gradients. 

First molecular genetic studies to characterize the communities of phototrophic purple bacteria were based on sequences of the *pufM* gene and revealed a remarkable high diversity in different habitats [66,67,68]. Most sequences retrieved from a frozen Antarctic lake represented a diverse array of sequences related to the Betaproteobacteria *Rhodoferax* and *Roseateles* (a bacteriochlorophyll-containing aerobic bacterium) and to the aerobic bacteriochlorophyll-containing Alphaproteobacteria *Acidiphilium* and *Bradyrhizobium* [68]. The high diversity and abundance of phototrophic bacteria related to the aerobic anoxygenic phototrophic *Roseobacter* and to the facultative aerobic anaerobic phototrophic *Rhodobacter/Rhodovulum* clades in the open oceans was subject of a number of metagenomic studies and supported the high abundance and great diversity of this group of phototrophic bacteria in ocean waters [44,69]. Their abundance and wide distribution was substantiated in the Red Sea and the Mediterranean Sea [70,71], in shrimp ponds in Thailand [72] and in Delaware estuary [73]. Most of these studies used separation of environmental amplicons on DGGE gels and sequencing of the separated bands to demonstrate the diversity of the *pufM* gene. However, due to the limitations of separation of bands on the gels and the short sequence fragments (<228 nt) considered, these studies lacked sufficient phylogenetic resolution to identify species or even genera. Nonetheless, they have demonstrated the presence of an immense biodiversity of aerobic phototrophic bacteria in marine habitats, which is to a great part not represented by cultured bacteria and in part not even significantly related to known bacterial species [45,71]. 

Remarkable are the studies on Soap Lake (Washington), which is a small soda lake with a distinct chemocline (at 20 m depth), a maximum depth of 24 m and high concentrations of sulfide reaching up to 175 mM near the sediment [74]. Both culture-based and culture-independent approaches were used to study the community of phototrophic bacteria in this lake. With the exception of a strain related to the purple nonsulfur Alphaproteobacterium *Rhodobaca bogoriensis,* several purple sulfur bacteria (representatives of *Ectothiorhodospira,* a strain related to *Thiorhodopsira sibirica* and *Thiocapsa imhoffii*) were isolated [74]. Though the same primers as in a previous study on an Antarctic lake [68] were used, no aerobic phototrophic bacteria, but primarily purple sulfur bacteria (Gammaproteobacteria) were found in *pufM* amplicons from environmental DNA. This is in accord with the expected presence of bacteria specifically adapted to the highly sulfidic and also alkaline environment. Sequences from a total of 12 bands (136 nt long) of *pufM* amplicons could be obtained after reamplification of bands from DGGE [74]. Only two of these were identical to one of the isolates from Soap Lake, to *Thiocapsa imhoffii*, which apparently is the dominant purple sulfur bacterium at the chemocline, while others showed only distant relations to *Rhodoferax*, *Rhodocyclus* and *Ectothiorhodospira* [74]. In conclusion, only a small part of the diversity of phototrophic purple bacteria of this extreme habitat was brought into culture and a better resolution of the phylogenetic diversity will probably be obtained with primer systems targeting the complete sequence of *pufLM* genes. 

Systematic studies with higher phylogenetic resolution and the species-specific analysis of complex environmental communities of phototrophic purple bacteria were enabled by the application of a primer pair that amplified sequences of both *pufL* and *pufM* genes (approx. 1400 nt) of most purple bacteria. With these primers, a comprehensive database using the almost complete sequences of most cultured purple sulfur bacteria was established and their phylogenetic relationship was verified [48]. The established phylogeny of the *pufLM* genes allowed confident identification of species and phylotypes in environmental samples. Two case studies of salt lakes in the Chilean highland and of a coastal lagoon of the Baltic Sea (Germany) may highlight the possibilities of this approach for community studies [9,75].

### 5.6. Baltic Sea Coastal Lagoon

The phototrophic bacterial community of a brackish water Baltic Sea coastal lagoon was characterized on the basis of almost complete *pufLM* gene sequences and the impact of changes in temperature and salinity were determined under controlled conditions in the laboratory by using RFLP, cloning and sequencing [9,48]. This lagoon represents a habitat typical of many studies on phototrophic bacteria and quite a number of available cultures of phototrophic bacteria have been isolated from similar habitats [32]. Accordingly, major *pufLM* phylotypes of the community of purple sulfur bacterial of this brackish water lagoon affiliated to genera and species of phototrophic purple sulfur bacteria typically isolated from such habitats including *Marichromatium*, *Thiocystis*, *Thiorhodococcus*, *Allochromatium*, *Thiocapsa*, *Thiorhodovibrio*; but some sequences were related to moderately halophilic *Halochromatium* and *Thiohalocapsa* species [32,76,77,78]. Quite important, at least 5 out of 20 identified phylotypes of purple sulfur bacteria could be clearly assigned to a known species, 10 additional phylotypes to a genus and only 5 phylotypes had sequence similarities (83.4%–85.6%) slightly below the proposed limit of 86% *pufLM* sequence similarity to the closest known type strain and therefore might be regarded as representing new genera [9]. Thus, the purple sulfur bacteria in the lagoon more or less are known at the genus level but novelty of these bacteria is high at the species level.

**Figure 3:**
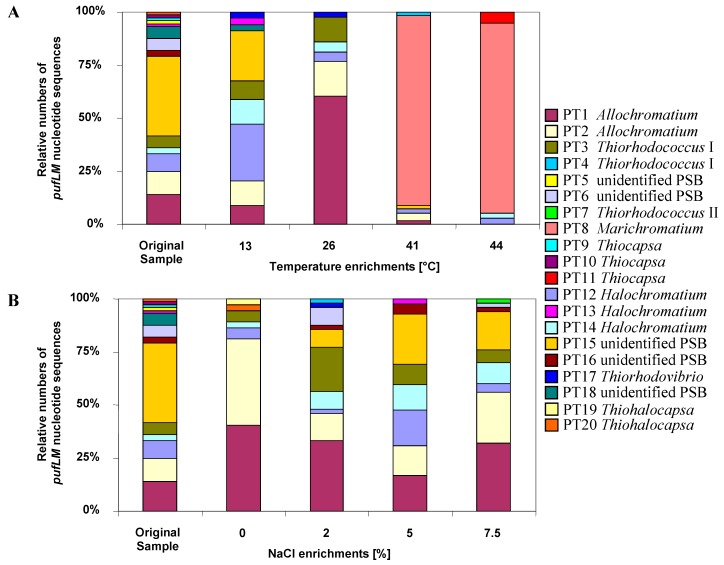
The contribution of different phylotypes to the community of purple sulfur bacteria (PSB) in a Baltic Sea lagoon is shown. Twenty phylotypes were identified in original sample and under different enrichment conditions on the basis of *pufLM* gene sequences. The figure depicts the composition in the sample and under the influence of different temperatures and salt concentrations (from [9]).

Both temperature and salinity had a significant influence on the community structure (Figure 3). In experimentally set up gradients of temperature (13–44 °C) and salinity (0%–7.5% NaCl) in identical culture media suited for purple sulfur bacteria, the community of purple sulfur bacteria was characterized by sequence analysis of *pufLM* clone libraries and compared to that from the natural habitat. The highest diversity of identified phylotypes was observed in the natural sample (23.5 °C, 2% salinity). Most phylotypes in the habitat were tolerating the complete range of salt concentrations and developed up to 7.5% NaCl. While the community at 0% NaCl was clearly dominated by only two *Allochromatium* phylotypes, the community was more divers at all other conditions [9]. With the exception of three phylotypes found as single clones in the environmental sample, all were retrieved at least from one of the enrichments. In addition, six phylotypes of purple sulfur bacteria were retrieved only after enrichments, but not found in the environmental sample. Among these were phylotypes most similar to the type strains of *Thiorhodococcus mannitoliphagus* (99.8%), *Thiorhodococcus kakinadensis* (98.2%), and *Marichromatium gracile* (100%). This is quite remarkable and indicates an even higher diversity in the environmental sample than resolved by its metagenetic analysis and also demonstrates that media and culture conditions were quite appropriate for almost all purple sulfur bacteria recognized in the environmental sample by the genetic approach.

An interesting property of *Marichromatium gracile*, which has been repeatedly isolated from marine coastal habitats [32,62], was demonstrated by these experiments. A dramatic shift in the community composition occurred at elevated temperatures of 41 and 44 °C when a single phylotype of *Marichromatium gracile* became most prominent, which was not detected at lower temperatures and not in the habitat sample. The clear preference of *Marichromatium gracile* for elevated temperatures points to its obvious competitive advantage in the shallow-water habitats if heated during daytime by the sun and supports findings of Serrano *et al.* who characterized a slightly thermophilic strain of this species [79].

### 5.7. Salt Lakes of the Salar de Atacama

The salt lakes of Chilean Salares represent an extraordinary and extreme habitat with special conditions regarding salt concentration and composition, irradiation and drastic diurnal changes in temperature [80,81]. Anoxygenic phototrophic purple bacteria, including a diverse group of aerobic phototrophic bacteria of the so-called *Roseobacter/Rhodobacter* clade are common to these lakes [82]. Like other hypersaline environments, some shallow lakes of the Salar de Atacama (Laguna Chaxa and Laguna Tebenquiche) exhibit extended purple-red colored microbial mats in and on the sediment surface. A comprehensive study of environmental communities of anoxygenic phototrophic purple bacteria of these salt lakes using the advanced *pufLM* primer system of [48] with focus on purple sulfur bacteria demonstrated highly diverse and variable communities of these bacteria [75]. The great majority of purple sulfur bacterial phylotypes of this habitat could be related to known purple sulfur bacteria, but was supposed to be new at the genus level or even at higher taxonomic rank [75]. 

The communities of purple sulfur bacteria from both salt lakes were characterized by the presence of representatives related to the type strains of moderately and extremely halophilic *Chromatiaceae* such as *Halochromatium salexigens*, *Halochromatium glycolicum*, *Thiohalocapsa halophila, Ectothiorhodospira mobilis, Ectothiorhodospira variabilis* and *Halorhodospira halophila* as closest relatives [76,77,78,83]. Evidence was also obtained for the presence of several phylotypes of BChl *b*-containing anoxygenic phototrophic bacteria distant to (<80% sequence similarity) the genera *Thiococcus*, *Thioflavicoccus* and *Thioalkalicoccus*, that form a separate phylogenetic branch among the purple sulfur bacteria [48,52,84]. These bacteria are known as inhabitants of marine sediments and have a particular advantage in sediment habitats due to their special pigment content with *bchl-b* [85,86,87]. Therefore, their presence in these salt lake sediments is not surprising, though they have been rarely isolated from salt and soda lakes [84]. 

Only two out of 24 phylotypes identified as phototrophic Gammaproteobacteria could be clearly identified on the species level (*Ectothiorhodospira mobilis* and *Thiohalocapsa halophila*), whereas the great majority of phylotypes had a *pufLM* sequence of such a low similarity (<80%) to known purple sulfur bacteria that quite likely they might represent new genera. Most remarkable was the dominance and diversity (11 phylotypes) of a novel, so far unknown lineage of *pufLM* containing Gammaproteobacteria, which was highly diverse and prevalent in different lakes of the Salar de Atacama [75]. 

Phototrophic Betaproteobacteria are rare in the salares of the Altiplano, but occasionally single clones of a Betaproteobacterium only distantly related to *Rubrivivax* [75] and others related to *Rhodoferax antarcticus* [82] were identified.

In conclusion and in contrast to the situation in the coastal Baltic Sea lagoon, most of the purple sulfur bacteria recognized by *pufLM* sequences in these salt lakes represent new bacteria at the genus level or higher taxonomic rank. This depicts quite well the extraordinary situation of the habitats in the Chilean highlands with extreme climatic and environmental conditions and the great geographic distance to all so far investigated habitats of phototrophic bacteria and points out the uniqueness of their bacterial communities.

### 5.8. Biodiversity of the bchY Gene

Another promising approach to analyze the diversity of phototrophic bacteria used the sequence of the *bchY* gene, which is present in phototrophic bacteria containing either a type I or a type II photosystem and was demonstrated to provide amplification products from various phototrophic purple bacteria, green sulfur bacteria and also green nonsulfur bacteria [88]. The *bchY* gene encodes the Y subunit of chlorophyllide reductase, which is at a branch point in the biosynthesis of chlorophyll and bacteriochlorophyll [89]. It is the only enzyme of this pathway present in all known anoxygenic phototrophic bacteria, but absent in oxygenic phototrophs. Therefore, genes coding for this enzyme should be good candidates for targeting the bacteriochlorophyll-containing anoxygenic phototrophic bacteria. A degenerate primer set for this gene was elaborated and primer specificity and coverage were evaluated using both *in silico* and *in vitro* techniques. The primer set was applied to *bchY* sequences present in databases and to pure cultures and environmental populations of anoxygenic phototrophic bacteria [88]. It was suggested that these primers have a broad applicability to study environmental communities of the phototrophic bacteria [88], though the sequence information retrieved is much lower compared to the more specific target genes *fmoA* and *pufLM*. Expected length of PCR products of the *bchY* gene are 480 bp for green sulfur bacteria, green non-sulfur bacteria, and *Heliobacteria* and 510 bp for phototrophic purple bacteria [88]. With this primer set the diversity of phototrophic bacterial communities was analyzed in Lake Kinneret and in the Mediterranean Sea [88]. Although this approach appears promising to cover all anoxygenic phototrophic bacteria with a single primer set, extended studies on environmental samples and in particular a comprehensive database with sequences from all cultured anoxygenic phototrophic bacteria still is lacking. When the phylogeny of this gene will be established with the available type strains of phototrophic bacteria, the *bchY* gene may prove to be an additional tool to systematically analyze natural communities of phototrophic bacteria.

## 6. Oxidation and Reduction of Sulfur Compounds

Sulfur compounds play important roles in energy generation of microorganisms, either as final electron acceptor in respiratory processes (e.g., sulfate and sulfur respiration) or as electron donors for energy generation in chemo- and photolithotrophic ways of life. The cycling of sulfur compounds by oxidation and reduction processes is governed by a number of prokaryotes from different phylogenetic lineages that use different biochemical pathways. In marine sediments sulfate reduction is a major process in organic matter degradation [90,91] and produces sulfide, which in turn is an important electron donor for sulfur-oxidizing bacteria. Though sulfate is the most abundant electron acceptor for anaerobic bacterial respiration in the ocean, the primary niche of sulfate reduction is the anoxic marine environment and therefore sulfate reduction is largely restricted to the anoxic zone. The primary ecological niche of chemotrophic sulfur oxidizers is the oxic/anoxic interface of stratified environments where oxygen and sulfide co-occur, though nitrate-reducing sulfur oxidizers and phototrophic sulfur bacteria specifically thrive in the anoxic environment.

Different biochemical pathways are involved in the sulfur cycling. One of the pathways for oxidation of sulfur compounds is the well-known reaction sequence including the formation and oxidation of adenosine-5′-phosphosulfate (APS) as intermediate with the involvement of disulfite reductase and APS reductase as key enzymes. First important insight into the evolution of these pathways was obtained from the comparable analysis of enzymes involved in the dissimilatory reduction of sulfate and in the oxidation of reduced sulfur compounds. Key enzymes of sulfate-reducing bacteria are APS reductase and disulfite reductase and already Peck [25] concluded from properties of the involved proteins that these enzymes share common evolutionary precursors in sufur-oxidizing and sulfate-reducing bacteria. Highly significant sequence similarities of APS reductase (AprA) and dissimilatory disulfite reductase (DsrAB) from the phototrophic sulfur-oxidizing gammaproteobacterium *Allochromatium vinosum*, the sulfate-reducing deltaproteobacterium *Desulfovibrio vulgaris* and the sulfate-reducing archaeon *Archaeoglobus fulgidus* strongly supported the hypothesis that these systems are true homologues and common ancestral genes for dissimilatory sirohaem sulfite reductases as well as APS reductases exist [92]. Today we know that *Archaeoglobus* has gained a true bacterial *dsr* gene by lateral gene transfer presumably from a bacterial ancestor [19]. Suitable marker genes for profiling the communities of both sulfate-reducing bacteria as well as sulfur-oxidizing bacteria using this pathway are provided by the genes of APS reductase (*aprA*) and of the dissimilatory sulfite reductase (*dsrAB*) [20,92,93].

A second important and widely distributed pathway of sulfur oxidation is the so-called Sox-pathway, which includes complex reaction sequences [94,95]. The *sox*B gene encoding the sulfate thioesterase was established as a suitable marker gene for this pathway [21,96,97,98]. 

### 6.1. Adenosine-5′-Phosphosulfate Reductase (APS Reductase), aprA

The adenosine-5′-phosphosulfate reductase (APS reductase) is a key enzyme of microbial sulfate reduction and also of sulfur oxidation processes and is common to sulfate-reducing bacteria and archaea and also to an important group of sulfur-oxidizing bacteria. A modified primer pair targeting the *aprA* gene was applied to a large number of reference strains from sulfate-reducing and sulfur-oxidizing prokaryotes [20]. This study further demonstrated that the reductive- and oxidative-type APS reductases are highly conserved among sulfate-reducing bacteria (SRP) and sulfur-oxidizing bacteria (SOB) and that the applied primer system allowed the concomitant detection of both sulfate-reducing and sulfur-oxidizing prokaryotes, which form separate lineages [20]. These authors explicitly, state that “although affected by several lateral gene transfer (LGT) events involving representatives of SRP and SOB lineages, the *aprA* genes have in general been transmitted vertically during evolution, which supports their usage as functional gene markers in microbial diversity surveys”. By using this *aprA* gene-based approach, a high diversity of sulfur-oxidizers was detected and clear differences noted in communities of the studied habitats with putative chemolithoautotrophic sulfur oxidizers of Beta- and Gammaproteobacteria being dominant in the hydrothermal sphere while sulfur-oxidizing Alphaproteobacteria were the major component in non-hydrothermal samples [20]. The same authors demonstrated the presence of phylotypes related to five sulfur-oxidizing alphaproteobacterial and gammaproteobacterial species and one sulfate-reducing archaeon in the microbial community associated with the Caribbean deep-water sponge *Polymastia corticata* by comparing *dsr*, *apr* and 16S rRNA gene sequences [99].

Also deep-sea subseafloor communities of sulfur-oxidizing and sulfur-reducing microorganisms from sediments of three sites of the Pacific margin off Japan revealed a markedly diverse community of *aprA* sequences [100]. 135 distinct phylotypes were recognized from a total of 692 *aprA* gene clones. Though composition and diversity varied largely between the sites and with the sediment depths, Desulfobacteraceae in general represented the major group of sulfate-reducers in most samples, followed by Desulfobulbaceae in several and *Desulfotomaculum*-related phylotypes present only in a few samples [100]. At least eight clusters unrelated to known cultivated sulfate-reducers were identified in different abundance in the samples. Among two major lineages with 45 phylotypes of sulfur-oxidizing bacteria, Gammaproteobacteria were dominant and a larger number of them could not clearly be assigned to known taxa even at the family level, because of the lack of phylogenetically close *aprA* reference gene sequences [100]. 

Novel groups of sulfur-oxidizing Gamma- and Alphaproteobacteria were recognized in studies of marine intertidal sediments from the German Wadden Sea by analysis of the diversity of *aprA* and *dsrAB* gene libraries [101]. The phylogenetic relations of *dsrAB* sequences from this study revealed a large hidden diversity of so far unrecognized sulfur-oxidizers both of Alpha- and of Gammaproteobacteria, part of which was supposed to perform a chemoautotrophic way of life [101].

The analysis of sediments of the Black Sea and the deep biosphere of the Peru margin revealed that the sequences of *aprA* and *dsrA* present in the samples affiliated to corresponding phylogenetic clusters [102]. The authors concluded that both of the functional genes represent sulfate reducers of equal phylogenetic positions. This was particularly significant for *aprA* and *dsrA* clone libraries from the Black Sea sediment where almost all *aprA* and all *dsrA* sequences affiliated either to *Desulfobulbus elongatus* or to *Desulfotalea psychrophila* [102].

Altogether, these few investigations have demonstrated that *apr* gene sequences are valuable tools to analyze the environmental communities of sulfate-reducing as well as sulfur-oxidizing bacteria. In both groups in addition to *aprA* the sequences of *dsrAB* give additional significance to the analyses.

### 6.2. Dissimilatory Sulfite Reductase, dsrAB

The dissimilatory siroheme sulfite reductase is a key enzyme in sulfate reduction and catalyzes the six-electron reduction of sulfite to sulfide. It is operating in the reversed reaction in sulfur oxidation as well and was first characterized from the sulfur-oxidizing *Thiobacillus denitrificans* [103]. The enzyme is considered to be highly conserved and therefore well suited to specifically approach the diversity analysis of sulfate reducing and sulfur-oxidizing bacteria in the environment. A 1.9-kb fragment encoding most of the alpha- and beta-subunits of the enzyme could be amplified from all lineages of SRB with a single primer set and the phylogenies inferred from the *dsrAB* gene of analyzed reference strains were consistent with those from the 16S rRNA gene, including the archaeal sulfate reducer *Archaeoglobus fulgidus* [104]. This view was extended to the sulfur-oxidizing bacteria, also showing largely congruent tree topologies of *dsrAB* and 16S rRNA gene sequences [105]. Though the enzymes from both groups can be approached with identical primer sets, the enzyme of sulfur-oxidizing bacteria is phylogenetically clearly distinct from that operating in sulfate reducers and both form separate phylogenetic groups [19,22]. 

In a study of a microbial mat of the hypersaline Solar Lake using *dsr* sequence analysis to evaluate the diversity of SRB, a highly diverse population of sulfate-reducing bacteria was found [105]. Special attention was paid in this study to the relationship of the sulfate-reducing community to oxygen within a microbial/cyanobacterial mat. The most striking observation was that the highest diversity of sulfate-reducers was found in the permanently oxic part of the mat and most of these sequences were closely related to members of the *Desulfonema* and *Desulfococcus* group. Though several SRB were known to be oxygen tolerant and some may have limited capacity to respire with oxygen [106], the high diversity and even dominance of *Desulfonema*-like SRB in the oxic part gave evidence for a possibly specific adaptation of these bacteria to life at the oxic/anoxic interface or even under microoxic conditions [105].

The analysis of *dsrAB* genes was also applied to study the diversity of sulfate-reducing bacteria in the Guaymas Basin [107], in which members of the Desulfobacteriales were identified, a larger number of which were related to *Desulfobacter* species. In addition, a new group of sulfate-reducers forming a cluster separate from all known sulfate-reducers was found that could not be identified in clone libraries of the 16S rRNA gene [107]. 

The sulfate reducing bacteria represent a good example to demonstrate the advances in our understanding of the biology and ecology of a microbial group made over the past decades. Until the 1970s these bacteria were regarded as a highly specialized group, strictly dependent on anoxic conditions and highly restricted to the metabolism of a few organic molecules only. The pioneering work of Fritz Widdel had initiated a new vision on the biology of these bacteria [108]. He cultivated and characterized a large number of new species and groups of sulfate-reducing bacteria and added new metabolic capabilities to them, such as autotrophic growth and complete oxidation of substrates and significantly extended the knowledge on the substrates used by these bacteria [108]. Our recent advances on their ecology primarily come from metagenetic and metagenomic analyses, which significantly increased our knowledge on the ecology of the sulfate-reducing bacteria. In particular their high diversity and the occupation of special ecological niches was demonstrated and specifically well resolved by the application of *aprA* and *dsrAB* as functional genes.

The advances achieved by functional gene studies over the past decade are well represented in the extensive work on *dsrAB* by Müller *et al.* [19]. In order to establish a comprehensive reference of *dsrAB* sequences, these authors constructed a consensus tree including more than 1200 sequences with approx. 1.9 kb length. Four major branches of *dsrAB* families were recognized: the reductive bacterial type, the oxidative bacterial type, the reductive archaeal type and a branch represented so far only by a second copy of this gene in *Moorella thermoacetica* [19]. With a few exceptions that are considered to indicate lateral gene transfer (LGT), it was confirmed that the phylogenies of 16S rRNA and *dsrAB* genes in sulfate-reducing and sulfur-oxidizing bacteria are largely congruent [19,22]. 

Among the reductive bacterial type of *dsrAB*, these studies established so-called superclusters, the Deltaproteobacteria, the Nitrospirae supercluster (previously named *Thermodesulfovibrio* supercluster), the Firmicutes group, and an environmental supercluster 1 [19]. The *dsrAB* sequences of the euryarchaeal *Archaeoglobus* formed a separate branch in this reductive bacterial-type *dsrAB* family tree [19]. The true archaeal type *dsrAB* sequences are represented by the genera *Pyrobaculum*, *Vulcanisaeta* and *Caldivirga*, though sulfate reduction so far has been demonstrated only for *Caldivirga maquilingensis*.

The oxidative *dsrAB* tree forms a monophyletic enzyme family that is phylogenetically distinct from all “reductive *dsrAB* sequences” and contains branches of Alpha-, Beta- and Gammaproteobacteria and Chlorobi. It is considered from the branching pattern of the tree that this branch evolved by functional adaptation from an ancient reductive *dsrAB*. With one significant exception (a single strain of *Thioalkalivibrio nitratireducens*, in contrast to other species of this genus), the sequence-based relationships of *dsrAB* are in good accordance with the 16S rRNA based phylogeny [19]. 

With this background, the species identification in environmental samples was attempted. A threshold of 90% *dsrAB* sequence identity (in general corresponding to <99% 16S rRNA gene sequence identity) was recommended to distinguish species. With this assumption the core dataset represented 647 species-level phylotypes of the reductive and 118 of the oxidative bacterial *dsrAB* type. A total of 299 environmental sequences of the core dataset did not affiliate with members of any described taxonomic family, but formed 13 stable, monophyletic lineages equivalent to the family level [19]. It has been approved that *dsrAB* sequences are useful phylogenetic markers to identify recognized taxa (genera/species) on the basis of environmental *dsrAB* sequences or in environmental metagenomes [19]. The large datasets of environmental sequences also gave rise to the analyses of habitat preferences and specific association of species and phylotypes to broad environmental categories [19]. 

### 6.3. Sulfate Thioesterase, soxB

Thiosulfate is an important and stable intermediate in the oxidation of reduced sulfur compounds and according to its central position within the biological sulfur cycle, it can undergo a number of transformations both in oxidative as well as reductive pathways. Among the oxidative pathways a central role has been given to the Sox enzyme system, which apparently is widespread among sulfur-oxidizing bacteria (SOB) and has primarily been discussed in regard to thiosulfate oxidation although it may be involved more generally in reactions of sulfur oxidation [94,95]. 

A key enzyme of the thiosulfate-oxidizing multi-enzyme complex is SoxB containing a prosthetic manganese cluster in the reaction center and being essential for thiosulfate oxidation by *Paracoccus pantotrophus*. The SoxB enzyme complex appears to be widespread among various phylogenetic groups of sulfur-oxidizing photo- and chemotrophic bacteria that oxidize thiosulfate to sulfate and is involved in two pathways of sulfur oxidation [21,94]: (i) The so-called *Paracoccus* sulfur oxidation pathway (PSO) catalyzes the oxidation of thiosulfate to sulfate without the formation of sulfur intermediates involving the SoxCD enzyme complex; (ii) The so-called branched pathway lacks the SoxCD complex and catalyzes the oxidation of reduced sulfur compounds to intermediate sulfur compounds with the characteristic deposition of intra- or extracellular sulfur globules [95,109,110]. The SoxB enzyme appears to be indispensable for both pathways. 

Based on the *soxB* gene, the phylogeny of the sox-pathways has been evaluated for use in biodiversity studies [21,96]. A primer system and PCR protocol for the detection of sulfur-oxidizing bacteria was developed to specifically amplify a *soxB* fragment of approx. 1000 bp from sulfur-oxidizing bacteria. The study of the primary and secondary structure of the amino acid sequences revealed, that the selected priming sites were highly conserved and had only a few amino acid changes among the available sequences [96]. The conserved regions were present in the same relative order and in identical regions of all *sox*B genes demonstrating an orthologous relationship of the genes from the various bacteria included in this study [96]. Sequences of *sox*B from a small number of reference strains, new isolates and environmental DNA from a hydrothermal vent habitat in the North Fiji Basin were compared and used to infer relationships of *sox*B between sulfur-oxidizing bacteria belonging to various 16S rRNA-based phylogenetic groups [96]. Though major phylogenetic lines derived from 16S rRNA genes correlated well with *soxB* phylogeny, indications of lateral gene transfer were also visible [96]. The general picture was marked by clearly separated branches of thiosulfate-oxidizing green sulfur bacteria and Proteobacteria as well as distant relationships between representatives of Alpha-, Beta- and Gammaproteobacteria including species of *Halothiobacillus, Thiobacillus, Starkeya.*

In a study with more than 120 sulfur-oxidizing bacteria, the wide distribution of the *soxB* gene in different phylogenetic groups of phototrophic and chemotrophic SOB was confirmed [21]. Though indications of lateral gene transfer were recognized, the lineages according to 16S rRNA-derived phylogeny could be clearly recognized in the *soxB* phylogeny [21].

An interesting case study alongside geochemical gradients of the outflow of Rattlesnake Spring (Oklahoma) demonstrated the spatial distribution of *soxB*-based phylotypes, which correlated well with environmental gradients [111]. It could be demonstrated that *soxB* gene sequences varied with the geochemical conditions. Sequence differences of the *soxB* gene were found to correlate to the utilization of the branched or the PSO Sox pathway and it was concluded that the spatial distribution of the different *soxB* gene variations along the outflow channel at Rattlesnake Spring could indicate niche partitioning among the bacteria that oxidize reduced sulfur compounds [111]. Some phylotypes could be assigned to genera (and species) using the branched pathway (*Thiobacillus, Thiomonas, Chlorobium, Allochromatium, Thiothrix, Halothiobacillus*) and others to genera using the PSO pathway (*Rhodovulum, Paracoccus, Bradyrhizobium*) [111]. Specific groups of sulfur-oxidizers were associated with different locations alongside the outflow channel: A succession was seen starting from a solid mat of *Thiothrix* almost as sole component, followed by varying abundances of the different members of the community downside the channel starting with major sequence numbers of *Halothiobacillus* and *Thiobacillus* and ending with a community dominated by *Rhodovulum*, *Bradyrhizobium* and *Thiobacillus* phylotypes [111].

Studies on the abundance and diversity of SOB populations in four Qinghai-Tibetan lakes based on *soxB* gene analyses revealed that salinity played a key role in controlling the diversity and distribution of SOB populations [112]. In freshwater Erhai Lake and low-salinity Gahai Lake 1, the SOB populations were dominated by Betaproteobacteria, whereas in the hypersaline Lake Gahai 2 and in Xiaochaidan Lake, the SOB populations were dominated by Alphaproteobacteria [112]. 

The diversity of the *sox*B gene in pure cultures of various halophilic and haloalkaliphilic sulfur-oxidizing bacteria (SOB) and environmental samples of salt and soda lakes in southwestern Siberia and in the Wadi Natrun (Egypt) was studied by Tourova *et al.* [113]. Though some of the *soxB* phylotypes were well related to sequences from known sulfur-oxidizing Gamma- and Alphaproteobacteria and also to isolates from the investigated lakes, others represented new uncultured lineages and were significantly distant from those present in pure cultures. These may represent new taxa at least at the species or genus level [113]. Most significantly, clearly different communities were found in the neutral salt lakes and in the soda lakes. In the neutral salt lakes, representatives related to *Thiohalorhabdus* and phylotypes of Alphaproteobacteria related to *Roseinatronobacter* and *Roseobacter* were found in one and phylotypes of *Marinobacter* and *Halothiobacillus* species in another one. In contrast, phylotypes belonging to the *Thioalkalivibrio* cluster were common to the soda lakes and represented major or single phylotypes in two of them (including a mix of samples from different soda lakes of the Wadi Natrun). The third soda lake was dominated by a phylotype related to *Thiohalomonas denitrificans* and in addition showed representatives related to *Thioalkalibacter*, *Halochromatium,* *Thioalkalivibrio*, and to the alphaproteobacterium *Roseinatronobacter* [113]. Though *Halorhodospira* species are well known as dominant members of sulfur-oxidizing phototrophic bacteria in these lakes [114,115,116,117], sequences of these bacteria could not be detected in this study. This failure was explained by Tourova *et al.* [113] to be caused by the lack of recognition of the binding site of the primers. However, *soxB* was successfully amplified from close relatives of the genus *Ectothiorhodospira* [21] and therefore an alternative explanation could be the heterogeneity of the habitat and the absence of significant numbers of these bacteria from the analyzed samples. [Note: It appears likely that the heterogeneity of habitats in many studies is underestimated and not considered in the sampling protocols. Thus, the molecular analysis of a particular sample may not necessarily represent the community of the habitat, but of a particular, possibly not defined niche or a mixture of niches.] Altogether, this study clearly approves the highly selective nature of different environmental settings for shaping environmental communities of sulfur-oxidizing bacteria. The highly specific association of different phylotypes with different lakes and in particular the clear distinction between communities of soda lakes and of neutral salt lakes is an important result. The presence of a number of new lineages, not identical to known species and genera, demonstrates that these lakes harbor specific communities and have a large so far undiscovered diversity of sulfur-oxidizing bacteria. 

## 7. Denitrification

Denitrifying bacteria are a highly diverse group of facultative anaerobic bacteria and are able to switch from aerobic respiration with oxygen as terminal electron acceptor to anaerobic respiration with nitrate, nitrite and nitrogen oxides as terminal electron acceptors. Several key enzymes involved in the respiratory pathway of dissimilatory nitrate reduction are characteristic for the denitrification process and may qualify for analysis of denitrifying bacterial diversity. Most attention has been paid to a membrane-bound (*nar*) and a periplasmic nitrate reductase (*nap*) and two types of nitrite reductases (*nirK* and *nirS*). The search for suitable primer binding sites in genes involved in the denitrification process led to the construction of specific primers for *narH*, *narG*, *napA, nirS* and *nirK* and a more detailed study using the *narH* gene [118].

### 7.1. Dissimilatory Nitrate Reductases, narH, narG and napA

The membrane-bound nitrate reductase catalyzes the first step in nitrate reduction. A detailed analysis of the secondary structure of NarH revealed several conserved parts of the membrane-bound nitrate reductase ß-subunit (containing four iron-sulfur clusters as prosthetic groups which are fixed in the protein by four cysteine binding motifes) as possible priming sites for amplification and made *narH* (in comparison to *narG*, *napA, nirS* and *nirK*) to the preferred gene for biodiversity studies [8]. The specificity was approved by application of the primers to reference species including upcoming new gene sequences from the data bases and to new denitrifying isolates. Two primer pairs were successfully applied (Table 1), their specificity was demonstrated and the phylogenetic relationship of the corresponding gene sequences was compared to the 16S rRNA-based phylogeny [8]. Major phylogenetic groups as known from 16S rRNA-based phylogeny were recognized by *narH* sequences. The Firmicutes with *Bacillus*, *Staphylococcus* and *Paenibacillus* formed a distinct cluster, as did the Enterobacteria (*Escherichia, Serratia, Kluyvera* and *Serratia*), the Betaproteobacteria (*Ralstonia* and *Burkholderia*) and the Gammaproteobacteria (*Pseudomonas*). These results were a strong motivation for further application of the *narH* gene in environmental studies of denitrifying bacteria and to consider the *narH* gene as primary target for monitoring diversity and activity of denitrifying bacteria.

In a first case study using these *narH* primers, valuable insight into denitrifying communities in different parts of the Baltic Sea was obtained [8,118]. Several locations of the Baltic Sea were studied by a combined approach: in parallel, denitrifiers were isolated and identified from the sediments and the diversity of denitrifiers was studied by *narH* sequence analysis in the environmental metagenome using an advanced double gradient denaturing gradient electrophoresis (DG-DGGE) combined with sequencing of the separated bands from the DGGE gels. Well resolved gradient gels nicely demonstrated, that over an annual time period (samples analyzed in a monthly interval) at a reference station in the Baltic Sea (“Boknis Eck”) the community of nitrate reducing bacteria was quite stable at the sediment surface but showed great variation in the bottom-near water [118].

A clear stratification was seen in the denitrifying communities with different populations in the upper (1–3 cm depth) and lower (3–5 cm depth) compartments of the top sediment layers of the Kiel bight (Boknis Eck and Kiel Bight) and the Gulf of Finland. In these sediments a clear maximum of bands of *narH* sequences correlated well with a minimum in the nitrate concentrations and a maximum in the activity of nitrate reduction alongside the depth profile (from 2–4 cm depth) [118]. Significantly, none of the sequences obtained from the excised bands of DGGE gels was closely related to known sequences and cultured denitrifiers. Though representatives of *Bacillus* and *Pseudomonas* represented approx. 60% of the isolated denitrifiers from Kiel Bight, none of these were found in the environmental sequences [118]. Part of the sequences was associated with Betaproteobacteria, others with the Gammaproteobacteria, but nitrate reducing Alphaproteobacteria were not found in the sediments from Kiel Bight and Gulf of Finland. 

In contrast to the two sediments of shallow areas of the Baltic Sea, other sites located in the deep Gotland Basin (at 240 and 190 m depth) were characterized by anoxic and sulfidic water bodies above the sediments. Also in these sediments, the nitrate profiles had clear minima between 2–4 cm depth and most of the bands of nitrate reductase were found in these layers. However, all sequences retrieved from the DGGE gel bands were associated with Alphaproteobacteria and formed clusters in some distance to the denitrifying *Paracoccus denitrificans* [8].

It was concluded that different environmental conditions select different communities of nitrate reducing bacteria in upper and lower sediment layers below the oxic water bodies (Kiel Bight and Gulf of Finland) and below the almost permanent anoxic water bodies in the Gotland Basin. It was suggested that a possible coupling between nitrification and denitrification in the transition between oxic and anoxic parts of the stratified sediments selects specific groups of denitrifiers different from those that predominate in the deeper anoxic parts of these sediments, while the supposed competition between sulfate reducers and nitrate reducers governs the development in the mostly anoxic and sulfidic sediments in the deep Gotland Basin [8]. This conclusion was supported by the phylogenetic position of the cluster of nitrate reducers in relation to *Paracoccus denitrificans* and the assumption that these bacteria would be able to link sulfide and sulfur oxidation to nitrate reduction as is known from *P. denitrificans*.

Gene sequences of the α-subunit of nitrate reductase *narG* were applied for studies on nitrate reducing bacterial communities in sediments of a fresh water lake [119]. Similar to the study with *narH* the majority of the sequences from environmental DNA were related to Gammaproteobacteria while a number of Gram-positive bacteria were isolated from the sediments.

A periplasmic nitrate reductase encoded by *nap* genes is an alternate widely distributed enzyme of dissimilatory nitrate reduction and is expressed in the presence of nitrate. The core part of this enzyme is encoded by *napA* and is highly conserved in chemoautotrophic nitrate-reducing Epsilonproteobacteria [120]. Epsilonproteobacteria from different vent sites mediate Nap-catalyzed respiratory nitrate reduction but do not employ the membrane-bound Nar-system, and all *napA* genes from the vent sites were from Epsilonproteobacteria [120].

### 7.2. Dissimilatory Nitrite Reductases, nirS and nirK

Much emphasis has been given to the application of genes coding for nitrite reductases in denitrifying bacteria. In general, denitrifying bacteria carry one of the two types of nitrite reductases, either a copper-containing enzyme encoded by *nirK* or a cytochrome-containing (cyt c and cyt d) enzyme encoded by *nirS* [121]. This offers the possibility to distinguish between two groups of denitrifying bacteria on the basis of the presence of *nirK* or *nirS*. 

Communities of denitrifying bacteria in a number of freshwater and marine habitats have been studied by applying the genes of nitrite reductases [122,123,124,125]. Clear shifts in the community structure could be established along oxygen gradients in the South Pacific and the Black Sea [126,127].

It should be mentioned, that *nirK* and *nirS* also are involved in aerobic microbial nitrification and anaerobic ammonia oxidation, respectively. Quite interestingly *nirK* genes (in some cases also the gene encoding for the large subunit of nitric oxide reductase *norB*) have been found in aerobic ammonia-oxidizing bacteria [134] and point to a potential role of nitrite reduction in the metabolisms of these bacteria. The topology of the *nirK* phylogenetic tree of nitrifying Betaproteobacteria corresponded to that of *amoA* and the 16S rRNA gene [128]. These results suggest that *nirK* sequences retrieved from the environment may include sequences from ammonia-oxidizing bacteria and that *nirK* has coevolved with *amoA* in these bacteria. Because *nirK* and *norB* sequences from *Nitrosospira* spp. were phylogenetically not clearly separated from those of denitrifiers, it was suggested that they have been subjected to lateral gene transfer [135]. 

For the involvement of *nirS* in anammox bacteria and *nirK* in AOA see the following paragraphs.

According to T-RFLP analyses and gene sequences of denitrifying bacterial communities from Lake Kinneret, a monomictic freshwater lake in Israel, it could be demonstrated that different clades of bacteria using *nirK* appear to be adapted to different environmental conditions alongside the stratified water column, whereas such a specific adaptation could not be seen with bacteria carrying *nirS* [125]. A similar observation was made alongside a salinity gradient at Huntington Beach (California), with bacteria carrying *nirK* being associated to specific salinities, but those carrying *nirS* were not [136]. Another example supporting such differential adaptation to the environment between bacteria carrying *nirK* and *nirS* was reported from the suboxic zone of the Black Sea, where *nirK* sequences showed much greater variation with depth while *nirS*-bacteria were more homogenously distributed [127]. Apparently, denitrifying microorganisms carrying *nirK* show different responses to a variety of environmental conditions and occupy different ecological niches compared to those having *nirS*. It would certainly be interesting to learn more about the strategic differences of the two groups of denitrifiers.

## 8. Nitrification—Oxidation of Ammonia—*amoA*

The oxidation of ammonia is a key step in the global nitrogen cycle and ammonia-oxidizing microorganisms (AOM) are widely distributed in environments and have received a lot of attention over the past decades. Three important groups of microorganisms are contributing to the oxidation of ammonia: the aerobic ammonia-oxidizing bacteria (AOB), the anaerobic ammonia-oxidizing bacteria (anammox) and the archaeal ammonia oxidizers (AOA). The isolation and cultivation of all three groups in the laboratory are quite problematic, in particular due to their slow growth rates and poor growth yields. For decades, these circumstances severely limited progress of our knowledge in the biology of the AOB. After the recognition of archaeal ammonia oxidation, the major interest moved to ammonia-oxidizing archaea (AOA), to their role in the environment and to the relative importance of both groups in the nitrification process [17,137,138]. Only with the application of culture-independent molecular approaches was the recent progress in our understanding of the diversity and distribution of these microorganisms in the environment possible. 

### 8.1. Aerobic Ammonia-Oxidizing Bacteria (AOB)

Traditionally, ammonia oxidation was known as a property of the aerobic ammonia-oxidizing bacteria (AOB), which are typically chemoautotrophic bacteria. Based on 16S rRNA gene and *amoA* sequences, AOB are found in two monophyletic groups, one is belonging to the Betaproteobacteria with the genera *Nitrosomonas* and *Nitrosospira* (including *Nitrosolobus* and *Nitrosovibrio*) and the other belonging to the Gammaproteobacteria, including *Nitrosococcus oceani* and *Nitrosococcus halophilus* [139]. These bacteria oxidize ammonia to nitrite and the first step is catalyzed by a membrane-bound ammonia monooxygenase encoded by the *amoCAB* operon. The *amo* operon was found in multiple almost identical copies in Betaproteobacteria but in single copies in the Gammaproteobacteria of AOB [140]. 

The ammonia monooxygenase gene *amoA* turned out to be a suitable marker for the whole group of aerobic ammonia-oxidizing bacteria and *amoA* was applied to characterize representative environmental communities. A number of PCR primers to amplify *amoA* have been used, though considerable differences were found in their performance and specificity [125,141]. Many studies on the molecular diversity of AOB have made use only of a portion of the *amoA* gene as a molecular marker [129,141]. As the sequence stretch of *amoA* used is highly conserved and in addition is relatively short (around 450 bp), it provides less resolution than the 16S rRNA gene [142]. Indeed, many investigations on the diversity and community structure of AOB in a wide spectrum of environments have used the 16S rRNA gene as a target, which was possible due to the separate phylogenetic position of these bacteria. More recently, the whole *amoCAB* operon was used for molecular studies and a nested PCR approach was applied to amplify environmental *amoCAB* sequences, including the complete *amoA* gene and providing an increase of sensitivity in detecting *amo* genes in samples even with low abundances of AOB [130]. 

Apparently, AOB are also adapted to a variety of extreme environmental conditions. Sequences of *amo*A retrieved from an acidic hot spring in a Japanese gold mine revealed AOB highly similar to *Nitrosomonas* spp. [143]. AOB were also found in salt lakes of the Chilean Altiplano AOB by *amoA* and 16S rRNA gene sequence analysis of enrichment cultures from Salar de Huasco [80]. Several phylotypes related to *Nitrosomonas* were identified and sequences of *amoA* related to *N. halophila* and *N. nitrosa* were frequently found at all salinities. Ammonia-oxidizing Gammaproteobacteria were not found in Salar de Huasco. 

Studies on Mono Lake demonstrated that all *amoA* sequences affiliated with ammonia-oxidizing Betaproteobacteria, but Gammaproteobacteria and AOA could not be found [144]. Most of these sequences affiliated with sequences of *Nitrosomonas halophila* from other soda lakes, a Mongolian soda lake, and Qinghai Lake (China) and of a *Nitrosomonas* isolate from a Greenland alkaline tufa column. Others were related to *Nitrosococcus europaea* and *Nitrosomonas eutropha* [144].

### 8.2. Ammonia-Oxidizing Archaea (AOA)

Soon after the first discovery of *amo*-like genes in archaeal DNA from metagenomic libraries from seawater [17] and soil [137], *Nitrosopumilus maritimus* was isolated as the first ammonia-oxidizing archaeon [138]. This was followed by other ammonia-oxidizing archaea, e.g., the thermophilic *Candidatus* Nitrosocaldus yellowstonii [145] and *Candidatus* Nitrososphaera gargensis [146]. In fact, these organisms were so different from other known archaea that a new phylum was assigned to harbor these ammonia-oxidizing archaea and was named Thaumarchaeota [147]. Our knowledge on these AOA increased at a fast speed and several contributions have summarized this progress [131,148,149].

A number of primer sets have been developed to amplify archaeal *amoA* (examples are found in Table 1) [141,150] allowing the identification and quantification of AOA. A comparison of diversity studies based on the 16S rRNA gene and archaeal *amoA* from several environments indicate a substantial congruence in the phylogeny of crenarchaeal ribosomal and *amo* genes [23]. Using molecular approaches based on the amplification of archaeal *amoA*, AOA have been found in all types of environments, from freshwater to marine and hypersaline habitats, from coastal waters to the deep ocean and ocean sediments, as well as in terrestrial habitats and apparently are ubiquitous in marine, freshwater, and terrestrial environments [131,148]. It was realized that they are the major players in environmental ammonia oxidation. In their review on the physiology and diversity of ammonia-oxidizing archaea, Stahl and de la Torre wrote [149]: “The discovery of ammonia-oxidizing archaea (AOA), now generally recognized to exert primary control over ammonia oxidation in terrestrial, marine, and geothermal habitats, necessitates a reassessment of the nitrogen cycle. In particular, the unusually high affinity of marine and terrestrial AOA for ammonia indicates that this group may determine the oxidation state of nitrogen available to associated micro- and macrobiota, altering our current understanding of trophic interactions”.

Much of the progress on diversity and ecological distribution was coming out of the application of *amoA* sequence analysis. According to *amoA* sequences five clusters of AOA are recognized, the *Nitrosopumilus* cluster, the *Nitrosotalea* cluster, the *Nitrosocaldus* cluster, the *Nitrososphaera* cluster and the “*Nitrososphaera* sister cluster” [151]. They occur in extreme habitats such as hydrothermal waters from which *Nitrosocaldus yellowstonii* [145] and *Nitrososphaera gargensis* [146] were isolated. Using *amoA*, archaeal nitrification capability was detected in a series of terrestrial acidic hot springs of Kamchatka, Siberia and Iceland at temperatures between 82–97 °C [152]. Thaumarchaeal *amoA* and 16S rRNA gene sequences related to *Nitrosopumilus maritimus* have been recovered from different cold environments like mountain lakes [153,154] and deep-sea waters as well [155]. 

Although several sequences of archaeal *amoA* were retrieved from Salar de Huasco in the Chilean Altiplano and were found to be related to clone sequences from Qinghai Lake in Tibet and a drinking water system, AOA were not frequent in this salar [154]. In the oxic waters of Qinghai Lake, a saline soda lake in China, AOA were more abundant than AOB [153]. While the community of AOB was similar in the water and in the sediment, distinct communities of AOA existed in water and sediment [153].

Sequences of archaeal *amoA* also were recovered from *Aplysina aerophoba* and other marine sponges and AOA were found to form a sponge-specific subcluster and were considered to be part of a stable microbial community of sponges [156]. 

As shown by the analysis of *amoA* clone libraries, both AOB and AOA revealed great spatial variation along the Yangtze Estuary though the AOA had a much higher diversity. The community structure of both groups was correlated with several environmental parameters, but potential nitrification rates correlated well with the abundance of archaeal *amoA* but not with bacterial *amoA* [157]. 

In nitrifying Archaea (Thaumarchaeota) two variants of archaeal *nirK* (*anirKa* and *anirKb*) have been identified [132]. These variants showed different distribution patterns in the epi-, meso- and bathypelagic water column with *anirKa* being found throughout the water column while two different variants of *nirKb* predominated in the upper water layers and in the lower ones [132]. In addition, the community composition of *anirKb*, but not of *anirKa*, was highly similar to that of the archaeal *amoA* [132], which is pointing towards a common phylogenetic background of *nirKb* and *amoA*. Two distinct ecotypes of archaeal ammonia-oxidizers in the ocean were also recognized by using two *amoA* primer sets. One ecotype is adapted to high concentrations (1.2 µM in average) and the other to low concentrations (<0.01 µM) of ammonia [158]. This distribution pattern was supported on a geographical scale with dominance of the ecotype adapted to high concentrations of ammonia in Arctic and Antarctic epipelagic waters and of the ecotype adapted to low ammonia concentrations in low altitude ocean waters and in ocean environments [159], which is in accord with the different concentrations of ammonia in the oceanic provinces.

### 8.3. Anaerobic Ammonia-Oxidizing Bacteria (Anammox)

Another group of ammonia oxidizing bacteria is known that performs an anaerobic ammonia oxidation (anammox). These bacteria were identified as new unique Planctomycetes [160] and apparently are widespread in nature. Anaerobic ammonia-oxidizing bacteria have been found in a variety of habitats and are considered to play an important role in the nitrogen cycle. Due to their isolated phylogenetic position, 16S rRNA genes have been used in most studies of natural assemblages of anammox. These bacteria do not employ an ammonia monooxygenase in the oxidation of ammonia, but use a different pathway in which the oxidation of ammonia is combined with the reduction of nitrite and the production of dinitrogen [161]. The proposed pathway includes a nitrite reductase (*nirS*), a hydrazine synthase (*hzs*) and a hydrazine oxidase (*hzo*) but not an ammonia monooxygenase [162]. The *nirS* gene was used as molecular marker to study the presence and potential activity of anammox in the environment by RT-PCR [163]. Also the *hzo* gene was applied to identify anammox bacteria from marine sediments, coastal wetland, and the deep ocean [164]. In addition, the hydrazine synthase (*hzsA*) has been identified as a unique marker for studies of anammox bacteria and was applied to anammox enrichment cultures and various environmental samples including marine samples from the Barent Sea and Northeast Greenland [165]. These studies indicated a narrow phylogenetic width but a broad environmental distribution of anammox bacteria with a highly niche-specific community structure in different marine ecosystems.

## 9. Oxidation of Methane—Methane Monooxygenase *pmoA*

Aerobic methanotrophic bacteria perform an important ecological function in the global carbon cycle by oxidation and assimilation of methane. Much of our “early knowledge” from the 1970s to 1990s on these bacteria comes from studies in paddy soils and rice fields [26,166,167]. As large reservoirs of methane are present in the deep ocean as methane hydrates and methane escapes from cold seeps and hydrothermal vents, much methane is transported to the ocean waters and in part also into the atmosphere. Because methane is an important greenhouse gas, anaerobic as well as aerobic oxidation of methane has received much attention over recent years [24,168,169,170]. Early studies on methanotrophic bacteria have characterized these on the basis of a larger number of isolates and already established two distinct phylogenetic groups using either the ribulose monophosphate pathway (type I, methanotrophic Gammaproteobacteria) or the serine pathway (type II, methanotrophic Alphaproteobacteria) for carbon assimilation [166,167]. The first enzyme in methane oxidation of both pathways, the particulate methane monooxygenase is a key enzyme and its gene is well suited as a functional marker in studies on their diversity and ecological relevance. The phylogenies of the *pmoA* gene and 16S rRNA gene have been shown to be congruent and *pmoA* is well established as a phylogenetic marker gene for studies of methanotrophic bacteria in the environment [24]. It has also been known for a long time that the enzymes involved in oxidation of ammonia and methane, the ammonia monooxygenase and the methane monooxygenase share common catalytic properties and also evolutionary roots [26], which implies that primers for *amoA* and *pmoA* may amplify both genes. 

Primers of the *pmoA* gene have been frequently applied in environmental studies since many years, e.g., to study methanotrophic communities in Lake Washington [171], in rice field soil [18], in a biogas reactor [172] and in methane seeps of the ocean floor and other marine environments [133,173]. Indeed, primers that cover both methane and ammonia oxidizing bacteria revealed interesting ecological implications [18,26]. While ammonia-oxidizers related to *Nitrosospira* were amplified in an untreated soil, after the addition of methane a shift in the community favored the development of both type I and type II methanotrophic bacteria [18], which indicates clear functional differentiation of the two groups of bacteria and of the two enzymes in the environment.

Amplicon pyrosequencing techniques using *pmoA* sequences was applied to study the diversity of methanotrophic bacteria from environmental samples and from cultures of methane-oxidizing bacteria and to define phylogenetic groups and phylotypes [170]. Though the combination of culture-based diversity analyses with environmental diversity studies is a valuable approach in defining phylogenetic groups and phylotypes, for any systematic consideration the implication of pure cultures and defined type strains and species is required. In another comprehensive study, a large number of cultivated methanotrophic bacteria and of environmental *pmoA* sequences has been used to define major phylogenetic groups of methanotrophic bacteria, to refine the established grouping and to define habitat specificity at different systematic levels of these bacteria [24]. 

## 10. Biosynthetic Pathways of Deep Sea Hot Vent Microorganisms

Since their discovery in 1977 and first pioneering microbiological studies [174,175], hydrothermal deep sea vents have attracted microbiologists and were subject for a large number of studies on the diversity of microbial communities and on specific colonization of niches in this habitat [176]. One of the primary characteristics of these habitats is their dependence on chemoautotrophic microorganism as primary producers which form the basis of the food chain associated with these habitats. In the following, three studies will be highlighted in which multiple functional genes were used to study bacterial communities related to deep sea hydrothermal vents.

### 10.1. The Logatchev Hydrothermal Vent Field

The Logatchev hydrothermal field is an ultramafic-hosted system on the Mid-Atlantic Ridge, with the Irina II complex as main structure consisting of a large mound with several black smoker chimneys at the top and mussel fields surrounding the base of the chimneys [177]. Venting of diffuse fluids is an important process at this site. The fluids reach the seafloor with moderate temperatures and provide energy for free-living and symbiotic chemolithoautotrophic bacteria of dense populations of mussels and shrimps. 

**Figure 4:**
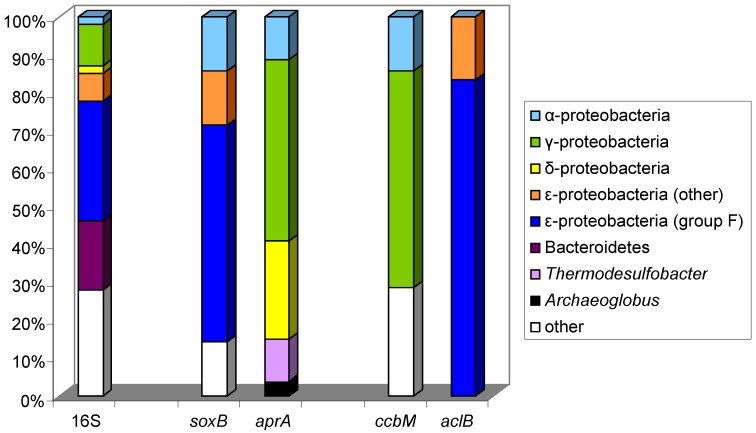
The microbial community composition of a hydrothermal fluid at a mussel field of Irina II at the Logatchev vent field is shown: on the left the phylogenetic groups according to 16S rRNA genes, the relative contribution of different phylogenetic groups to two sulfur oxidation pathways, the Sox-pathway (*soxB*) and the APS-pathway (*aprA*), in the middle and to two pathways of CO_2_-fixation, the reductive tricarboxylic acid cycle (*aclB*) and the Calvin cycle (*ccbM*) on the right (from [97]).

The microbial community structure within hydrothermal fluids from the Irina II structure of the Logatchev vent field was analyzed using genes involved in different pathways of sulfur metabolism (*soxB, aprA*) and autotrophic CO_2_-fixation by the Calvin-Benson-Bassham cycle (*cbbL*, *cbbM* genes) and the reductive tricarboxylic acid cycle (sequences coding for ATP citrate lyase *aclB*) as functional markers and the 16S rRNA gene as an additional phylogenetic marker [97]. For the first time, different key genes of important energy-yielding processes were analyzed together with marker genes of different carbon fixation pathways in order to unravel the metabolic properties of the chemolithoautotrophic primary producers at this deep sea vent site [97]. Dominant groups in the microbial communities of diffuse fluids from a mussel field were Epsilonproteobacteria (35% of the phylotypes) together with Gammaproteobacteria (17% of the phylotypes) and both groups performed sulfur oxidation and CO_2_-fixation [97]. As concluded from the tree topologies of *aprA*, *soxB,* *cbbM*, *cbbL* and *aclB,* both groups used different pathways for these processes (Figure 4) [97]. As *aprA* genes from sulfate-reducing bacteria can be clearly distinguished from those of sulfur-oxidizing bacteria [20], it was clear that part of the *aprA* gene sequences obtained could be assigned to sequences from sulfate-reducing Deltaproteobacteria. Sequences related to the genus *Desulfocapsa* were found in the 16S rRNA as well as in the *aprA* gene libraries. Among the lineage of *aprA* gene sequences belonging to sulfur-oxidizing bacteria, Gammaproteobacteria were clearly dominant (80%) and most of the sequences were similar to *aprA* sequences known from epibionts and endosymbionts from vent habitats [97].

Most of the *cbbM* sequences retrieved were related to sequences from Gammaproteobacteria and were most similar to those from *Thiomicrospira pelophila,* *Thiomicrospira thermophila* and to tubeworm symbionts including *Candidatus* Ruthia magnifica (the symbiont of *Calyptogena magnifica* [97]. These correlations can be taken as a clear indication that Gammaproteobacteria in the vent system are using the Apr system for sulfur oxidation and the Cbb system for carbon fixation and that the majority of these bacteria maybe related to symbionts or even engaged in symbiotic associations. According to phylogenetic analyses of the 16S rRNA, *aclB* and *soxB* genes, the Epsilonproteobacteria were the dominant group of bacteria in the hydrothermal fluids and sequences of the *aclB* gene were related to those of different groups of *Epsilonproteobacteria,* the great majority to the group F [97]. In addition, all *soxB* gene sequences that were retrieved were associated with this group. In conclusion, Epsilonproteobacteria are using the Sox-pathway for sulfur oxidation and are important primary producers in the vent system using the reversed tricarboxylic acid cycle [97]. It appears that Epsilonproteobacteria are of major importance as microbial players in sulfur oxidation and primary production in deep sea hydrothermal fluids and are well competing with other bacteria [178,179]. Considering energetic aspects they have clear advantages over the sulfur-oxidizing Gammaproteobacteria, because they use the energetically cheap reductive TCA cycle with less than half of the energy demand for CO_2_ fixation compared to the Calvin cycle used by the Gammaproteobacteria. Another important advantage explaining the dominance of these bacteria in deep sea hot vent ecosystems with steep environmental gradients is their metabolic flexibility, *i.e.*, anaerobic and/or microaerobic growth with reduced sulfur species and/or hydrogen as energy sources [178,179,180]. They also are able to utilize nitrate as an alternative electron acceptor to oxygen and quite interestingly Epsilonproteobacteria are the only group in hot vent communities employing the periplasmic nitrate reductase encoded by *napA* for this purpose [120].

### 10.2. Lamellibrachia Anaximandri

A functional gene study of endosymbionts of vestimentiferan tube worms from a hydrothermal vent site in the Mediterranean Sea identified as *Lamellibrachia anaximandri*, yielded interesting new insight into the functional pathways of the endosymbiont [181]. Genetic analyses indicated the presence of a single sulfide-oxidizing chemoautotrophic Gammaproteobacterium as endosymbiont [181]. However, the endosymbiont harbors genes of two different carbon fixation pathways, the Calvin-Benson-Bassham cycle (*cbbL*, *cbbM*) and the reductive tricarboxylic acid cycle (*acl*). Tubeworms of the genus *Lamellibrachia* are common to hydrothermal vent and cold-seep communities in the Pacific and other hydrothermal habitats. Comparison with tubeworm species (*Lamellibrachia luymesi*, *Escarpia,* *Seepiophila jonesi*) from cold seeps in the Gulf of Mexico revealed the presence of *acl* and *cbbL/M* genes in these species as well [181]. These results and also those reported for the endosymbiont of *Riftia pachyptila* [182] suggest that the presence of the two carbon fixation pathways might be common in vestimentiferan tubeworm endosymbionts, regardless of the habitat. The differential expression of these genes, the regulation of the two pathways and their activity remain open questions.

### 10.3. Rimicaris Exoculata

A multiple functional gene study was made with the epibacterial community associated with the shrimp *Rimicaris exoculata* which dominates the faunal biomass at some locations of deep-sea hydrothermal vent sites at the Mid-Atlantic Ridge [98]. This specialized epibiotic bacterial community located in the large gill chambers was studied in specimens from the Snake Pit hydrothermal vent field on the Mid-Atlantic Ridge by complementing a 16S rRNA gene survey with the analysis of genes involved in carbon (*aclB*, *ccbL, cbbM*), sulfur (*aprA*, *soxB*) and hydrogen (*hynL*) metabolism [98]. The community consisted mostly of Epsilon- and Gammaproteobacteria, but also contained a single phylotype of Deltaproteobacteria, closely related to the genus *Desulfocapsa.* Based on functional gene analyses, it was concluded that the Gamma- and Epsilonproteobacteria associated to the shrimp are capable of autotrophic growth by oxidizing reduced sulfur compounds, and that the Deltaproteobacteria as reducers of sulfate may enable the cycling of sulfur compounds in a microscale dimension within the gill [98]. The phylogeny of the epibionts based on the 16S rRNA gene was in most cases congruent with functional gene phylogenies, allowing the conclusion that the Epsilonproteobacteria associated with the shrimp could oxidize sulfur compounds via the Sox pathway and perform carbon fixation via the reduced TCA cycle. Most of these sequences were associated to Epsilonproteobacteria group F, known as the Thiovulgaceae, with *Sulfurovum lithotrophicum* as the closest cultured relative [98]. The Gammaproteobacteria could generate energy by oxidizing reduced sulfur compounds via the pathway involving Apr and Dsr and fix carbon via the CBB cycle. These sequences were related to other epibiont sequences and to *Leucothrix mucor* as the closest cultured bacterium [98]. Similar phylotypes of Epsilon- and Gammaproteobacteria have been identified in other epibiotic associations with hydrothermal vent invertebrates and they may be of general importance in consortia associated with such invertebrates [178,180,183]. In addition, genes of [NiFe] hydrogenase (*hynL*), which is involved in the oxidation of molecular hydrogen for energy generation, were identified. The relation of these genes to sulfate reducers and Epsilonproteobacteria highlights the potential of both groups to grow with hydrogen as an electron source. In relation to the genes of sulfur oxidation and carbon dioxide fixation it appears quite likely that in addition to Epsilon- and Gammaproteobacteria also the sulfate-reducing Gammaproteobacteria may contribute to autotrophic carbon fixation and productivity of the epibiotic community [98]. The location of these phylogenetic groups within the shrimp was confirmed by fluorescence *in situ* hybridization and this visualization supports the assumption of physiological interactions between the groups of sulfate-reducing and sulfur-oxidizing bacteria [98]. 

## 11. Conclusions

Molecular studies using functional genes as targets have not only presented important phylogenetic information of major biochemical pathways, but also gave crucial information on the competitive nature of the considered pathways in the environment, or rather their relevance for the competition of the organisms employing these pathways. Furthermore, detailed information on the functional diversity in many habitats and on the selection of and preference for specific habitats by microorganisms has accumulated in recent years. In this context, aspects of geographical distribution were also of major concern. Quite importantly, for most functional genes the general congruence with the phylogeny of the 16S rRNA gene has been established, and growing databases of functional gene sequences make it possible to trace their phylogeny and to identify species or phylotypes in the environment on the basis of functional gene sequences. 

Undoubtedly, a new era of microbial ecology was started with the introduction of 16S rRNA gene analyses in environmental studies and the established molecular microbial ecology opened up completely new possibilities to this research field. For the first time, microbial biodiversity studies were possible based on 16S rRNA gene sequences. A new dimension is given to microbial ecology with the introduction of functional genes as targets for biodiversity studies which enables the identification of strains and species on the functional gene level with the perspective of bioactivity studies under environmental conditions using functional gene transcripts.

### 11.1. Functional Diversity—The Competition of Pathways in Nature

One of the most significant findings resulting from the application of functional genes in environmental studies clearly points out the importance of biochemical pathways for performance and competition of microorganisms in the environment and for the selection of and preference for ecological niches by the microorganisms. In this context, the success of sulfur-oxidizing Epsilonproteobacteria in the gradient systems of deep sea hydrothermal vents is explained by the employment of energy efficient pathways such as the reversed TCA for carbon fixation and the Sox-pathway for sulfur oxidation as compared to the Calvin cycle and the APS pathway by sulfur-oxidizing Gammaproteobacteria [97,98]. Similarly, a clear advantage of AOA over chemoautotrophic AOB can be explained by higher affinity of AOA to ammonia and by employing the highly energy-efficient hydroxypropionate/hydroxybutyrate pathway for carbon dioxide fixation rather than the Calvin cycle [184]. It is amazing to see that the nitrite reductase genes *nirS* and *nirK* identify two groups of denitrifying bacteria with different strategies of environmental adaptation [125,126,127,136]. Furthermore, the phylogenetic analysis of these two genes sheds light on the evolution of anaerobic ammonia oxidation, in which *nirS* is involved, and on aerobic nitrification, in which *nirK* is involved [128,134,135]. A similar differential preference for ecological niches is also relevant for archaeal ammonia oxidizers and relates to the presence of *anirKa* and *anirKb* genes as well as to different variants of the archaeal *amoA* gene [132]. Although it would probably be much too simple to relate environmental competition to a single advantage and a number of different aspects should be taken into consideration, a single strong advantage might nevertheless be sufficient to explain success of the more efficient microorganism. Such an advantage could be the use of a highly efficient pathway of carbon fixation or the more efficient energy generation from a common energy source, but also a significantly higher affinity to a key substrate as outlined for several examples before. Certainly, over ancient times the evolution of pathways and the adaptation to environmental factors has caused dramatic changes in competition and replacement of microorganisms from their established niches by others more suited to this niche. Such an adaptation of strictly anaerobic sulfate-reducing bacteria to the oxic environment was witnessed by the application of *dsr* gene analysis in microbial mats of Solar Lake with a clear dominance of relatives of *Desulfonema* in this niche [105].

### 11.2. Environmental Conditions Determine Microbial Community Structure

Functional gene studies have convincingly and repeatedly demonstrated that small changes in the environmental conditions significantly can change the community composition and that individual species or phylotypes may have a narrow range of suitable conditions for development and successful competition. 

The application of *pufLM* and *fmoA* genes to the study of environmental communities of phototrophic purple and green sulfur bacteria has pointed out the relevance of salt relations and temperature responses to the distribution of species in the environment. A decent part of the environmental diversity of these bacteria is available in pure cultures and, depending on the habitat, a varying part of the diversity can be identified at the level of species and genera using *fmoA* or *pufLM* gene sequences. It was realized that coastal lagoons to a significant part harbor marine, salt tolerant and moderately halophilic species, most of which are known at the genus level and that in part drastic changes in the community composition occur as response to temperature and salinity changes. This can be taken as an indication that salt tolerance is an important property to adapt and survive in these habitats which can undergo large variations in salt content depending on evaporation and supply of new freshwater or seawater. In two Chilean salt lakes situated in close distance to each other (Laguna Chaxa, Laguna Tebenquiche), species and phylotypes were identified with the next relatives known to be specifically adapted to the high salt concentrations in the habitat (*Halochromatium*, *Thiohalocapsa*, *Ectothiorhodospira* and *Halorhodospira*). Despite this unifying aspect of the two salares, it was quite remarkable to see that even different samples from the same lake demonstrated a largely different composition of the communities of purple sulfur bacteria [75]. Thus environmental factors are highly selective for the development of species and very specifically determine the community structure also at a small scale. It is similarly striking to see the general differences of sulfur-oxidizing bacterial communities between alkaline soda lakes and other salt lakes, as analyzed by *soxB* sequences, and in addition to realize that a characteristic and typical community structure can be found in individual soda lakes and salt lakes [113]. 

Likewise, it is interesting to see that denitrifying Alpha- and Gammaproteobacteria, as demonstrated by *narH* gene sequences, occupy clearly different niches in strongly sulfidic marine sediments and in stratified sediments with an oxic surface layer. On top of this a clear preference for different denitrifiers is seen in different layers of the stratified sediment horizons [8]. Generally, it can be expected that alongside environmental gradients different microorganisms find their ecological niche and a succession of microorganisms can be observed. A good example of such a succession of sulfur-oxidizing bacteria is presented in the outflow of Rattlesnake Spring (Oklahoma), where the spatial distribution of *soxB*-based phylotypes alongside the environmental gradients was demonstrated [111].

### 11.3. The Challenges of Functional Diversity Studies in the Habitat

In view of the tremendous advances in molecular microbial ecology which was established only two decades ago, there is much enthusiasm in this research field. Nevertheless it must be considered that also the most advanced methods have at least some limitations. Although we are convinced that the use of specific functional primers for gene amplification gives the most comprehensive picture on the specific function’s diversity, we still have to consider biases, e.g., due to methods of DNA extraction from the environment and primer specificity. We also have to consider that a complete record of the species diversity may not be achieved, which is, however, more relevant to the analysis of complete metagenomes than to specific functional metagenetic analyses, no matter how accurate and extensive the sequence analyses may be (see the previous discussion to this aspect). In this sense, we have to accept that the true diversity of a given sample is higher than shown by the metagenetic and metagenomic analyses and that some species not seen under one condition may increase in importance or even become dominant under changed conditions. Such an incomplete coverage of environmental communities as well as the selective property of salt concentrations and temperature was nicely demonstrated in culture experiments with phototrophic purple sulfur bacteria from a coastal Baltic Sea lagoon using the photosynthetic *pufLM* genes [9]. In consequence, these findings highlight the immense diversity and flexibility of environmental communities and their potential to adapt to changes of the environmental conditions not only by metabolic flexibility of individual organisms but to a great part by changes in the community structure.

### 11.4. Selective Enrichment of Functional Groups

One of the oldest and strongest selective conditions employed by microbiologists is the use of specific culture media for enrichment and cultivation of various bacteria with different physiological demands [10]. The specificity of a particular enrichment and cultivation protocol always selects the same (or similar) species from different environmental samples, even if they are present in extremely minor portions (not detectable in metagenomes) and thereby verifies the wide distribution independent from the importance at the particular site of investigation. In fact, proper enrichment methods can be extremely selective such that they may enrich a species contributing only less than 1 cell per several billion. As an example, green sulfur bacteria could be enriched from oxic intertidal flats of the German Wadden Sea where they were present in estimated numbers of less than 1 cell per cm^3^, while the total number of prokaryotes in this sediment exceeded 10^10^ cells cm^3^ (Imhoff, unpublished). It is quite obvious that the analysis of environmental DNA of this sediment would not have given a sign of these green sulfur bacteria, as their abundance is far below the detection limit of these approaches (even using currently available high throughput sequencing techniques) and they are considered as an unimportant part of the community. However, whenever such sediments undergo environmental changes that allow the sulfide produced by sulfate-reducing bacteria to stay in the top layer of the sediment illuminated by the sun or even to penetrate into the water body of small lagoons, this minor portion of the community may rapidly form massive developments that are indeed often seen in suitable coastal habitats. The principal advantage of enrichment experiments is the high selectivity in regard to a particular functional property and this may be specifically used to determine the diversity of a given function, *i.e.*, anoxygenic phototrophic sulfur oxidation [9] or aerobic methane oxidation [18] either by *in situ* experiments under environmental conditions or in experimental enrichment studies in the laboratory.

Though we are far from being able to systematically describe the species diversity of natural communities, even of the well characterized anoxygenic phototrophic bacteria, results of the work discussed here demonstrate that progress is made with high speed and that this might well be achieved in the future. An important point to support this optimistic view is the fact that the combination of cultivation-independent and cultivation-dependent approaches demonstrated that most of the phylotypes seen *in situ* also maybe found after enrichment with culture conditions and media properly established, as exemplified for the phototrophic sulfur bacteria [9,185,186,187]. Similar experiments can be applied to any other physiological group and frame of physicochemical conditions, whenever the function is defined by a specific gene as target. Other examples where specific media and culture conditions have successfully been applied for the cultivation of specialized bacteria include extremely haloalkaliphilic phototrophic bacteria [115,116,117] and alkaliphilic chemotrophic sulfur-oxidizing bacteria [113]. As outlined before, the enrichment, cultivation and characterization of microorganisms from the environment remains an important part of ecological studies, which is indispensable to characterize the individual player in the ecosystem [188]. Increased efforts in cultivation approaches with various functional microbial groups are needed to improve the possible interpretation of metagenetic and metagenomic studies in the environment. 

For the future, the combination of *in-situ* meta-transcriptomic analyses and laboratory cultivation studies will provide deeper insights into the understanding of microbial communities and will add another new dimension to microbial ecology, the species-specific *in situ* measurement of activities in complex environmental communities. Activities of selected genes and dynamic changes thereof will enable analysis of the dynamic adaptation of communities to changes in the environmental conditions, including diurnal and long term changes, and relate these changes to the functional transcript and the phylotype. Functional gene studies as highlighted in this review are certainly forming the basis for such meta-transcriptomic studies.

## Figures and Tables

**Table 1:** Selected primers for functional gene analyses.

Gene	Biogeochemical Fucnction	Gene Product	Primer Name	Primer Sequence (5'-3')	Reference
*pufLM*	photosynthesis	photosynthetic reaction center	pufL67F	TTCGACTTYTGGRTNGGNCC	[48]
			pufM781R	CCAKSGTCCAGCGCCAGAANA	[48]
*fmoA*	photosynthesis	bacteriochlorophyll-a-protein	F-Start-fmo-modif	ATT ATG GCT CTN TTC GGC	[75]
			r-889-FMO	CCGACCATNCCGTGRTG	[47]
*bchlY*	photosynthesis	chlorophyllide reductase	bchY-fwd	CCNCARACNATGTGYCCNGCNTTYGG	[19]
			bchY-rev	GGRTCNRCNGGRAANATYTCNCCC	[19]
*soxB*	“sulfur” oxidation	sulfate thioesterase	soxB432F	GAYGGNGGNGAYACNTGG	[96]
			soxB1446B	CATGTCNCCNCCRTGYTG	[96]
*aprA*	“sulfur” oxidation	APS reductase	AprA-1-FW	TGGCAGATCATGATYMATGG	[20]
	sulfur reduction		AprA-5-rv	GCGCCAACYGGRCCTTA	[20]
*dsrAB*	“sulfur” oxidation	dissimilatory disulfite reductase	rDSR1Fa	AARGGNTAYTGGAARG	[22]
	sulfur reduction		rDSR1Fb	TTYGGNTAYTGGAARG	[22]
			rDSR1Fc	ATGGGNTAYTGGAARG	[22]
			rDSR4Ra	CCRAARCAIGCNCCRCA	[22]
			rDSR4Rb	GGRWARCAIGCNCCRCA	[22]
*narH*	nitrate reduction	dissimilatory nitrate reductase	narH50F	AARTGYATCGGYTGCCA	[8]
	denitrification		narH1040B	GTNCGRTYTCNGG	[8]
			narH403F	GGNCCNAACTGGGNGA	[8]
			narH403B	TCNTCCCAGTTNGGNCC	[8]
*nirS*	nitrate reduction	dissimilatory nitrate reductase	nirS1F	CCTAYTGGCCGCCRCART	[123]
	denitrification		nirS6R	CGTTGAACTTRCCGGT	[123]
*nirK*	nitrate reduction	dissimilatory nitrate reductase	nirK1F	GGMATGGTKCCSTGGCA	[123]
	denitrification		nirK5R	GCCTCGATCAGRTTRTGG	[123]
			Cunir3	CGTCTAYCAYTCCGCVCC	[128]
			Cunir4	GCCTCGATCAGRTTRTGG	[128]
*amoA*	ammonia oxidation	ammonia monooxygenase	amoA-1F	GGGGTTTCTACTGGTGGT	[129]
			amoA-2R	CCCCTCKGSAAAGCCTTCTTC	[129]
*amoCAB*	ammonia oxidation	ammonia monooxygenase	amoC58f	CTAYGACATGTCRCTGTGG	[130]
			amoB1179r	CCAAARCGRCTTTCCGG	[130]
*amoA*	ammonia oxidation	archaeal	Arch-amoAF	STAATGGTCTGGCTTAGACG	[131]
		ammonia monooxygenase	Arch-amoAR	GCGGCCATCCATCTGTATGT	[131]
*anirKa*		archaeal	anirKa-58F	ACBYTATTCGGAAGYACATACACA	[132]
		dissimilatory nitrate reductase	anirKa-579R	GYMATTCCGTACATKCCGGA	[132]
*anirKb*		archaeal	anirKb-61F	CTATTCGGARGTWCTTTYACTGC	[132]
		dissimilatory nitrate reductase	anirKa-555R	ACGTGTTGGTCCATTGCTGC	[132]
*pmoAC*	methane oxidation	methane monooxygenase	pmoC617f	ACACCTTCTGGTTCATGG	[133]
			pmoA682r	GAASGCNGAGAAGAASGC	[133]
*napA*	nitrate reduction	periplasmic nitrate reductase	NapV16F	GCNCCNTGYMGNTTYTGYGG	[120]
			NapV17R	RTGYTGRTTRAANCCCATNGTCCA	[120]
